# Facility-Based Assessment of Emergency Obstetric and Neonatal Care in Vanga Health Zone, Kwilu Province, Democratic Republic of Congo

**DOI:** 10.12688/wellcomeopenres.25137.1

**Published:** 2026-01-02

**Authors:** Mudji Junior, Kieran Desmond, Bill Sabwa, Mike English

**Affiliations:** 1 University of Oxford Nuffield Department of Medicine, Oxford, England, UK; 2 Health Services Unit, Health Services Unit, KEMRI-Wellcome Research Programme, Nairobi, Kenya, Nairobi, Nairobi, Kenya; 3 Institut National de Recherche Biomédicale, Kinshasa, Democratic Republic of the Congo

**Keywords:** Emergency Obstetric and Neonatal Care (EmONC), Maternal Health, Neonatal health, Health facility assessment, Quality of Care, Health system capacity, Democratic Republic of Congo, Resource allocation

## Abstract

**Background:**

Maternal and neonatal morbidity and mortality rates in the Democratic Republic of Congo (DRC) remain unacceptably high. The lack of empirical evidence on the capacity and performance of health facilities offering emergency obstetric and neonatal care (EmONC) is a central problem. With more real-world facility data, policymakers can make informed decisions on resource allocation, investment and harm-reduction strategies. This study collected data from 63 healthcare facilities in the Vanga Health Zone, Kwilu province, DRC to assess the quality of EmONC provision.

**Methods:**

a cross-sectional survey of all EmONC healthcare facilities excluding Vanga referral hospital in the Vanga Health Zone from September - December 2023. “Type A” facilities were defined as those employing doctors, “Type B” facilities did not employ doctors. Quality indicators measured were: infrastructure, equipment, supplies and medicines, human resources, EmONC signal functions and EmONC patient outcomes (caseload data was collected from two 3-month periods (Jan-March 2021 and Jan-March 2022)).

**Results:**

We identified widespread infrastructural deficiencies, including no water sources in 61/63 facilities, a complete lack of emergency transfer capability, and limited bed capacity. Stock inventories showed that 39/50 categories in type B facilities and 38/52 categories in type A facilities had “Poor” availability of basic EmONC equipment, supplies, and medications. The median number of nurses providing 24/7 care across all specialities in type B facilities was four. Doctors were employed at 5/63 health facilities (13 doctors total), none had received post-graduate training. Signal function data showed inadequate basic EmONC, plus widespread unsafe practices: caesarean-sections and blood transfusions performed without doctors, support or essential equipment. There were concerns over the validity of caseload data.

**Conclusion:**

Health facilities in Vanga Health Zone show inadequacies in all quality domains assessed and are unable to provide acceptable EmONC. We also present evidence of unsafe practices risking patient harm.

## Plain Language summary

Facility-based Assessment of Emergency Obstetric and Neonatal Care in Vanga Health Zone, Kwilu Province, Democratic Republic of Congo.

The DRC has one of the highest rates of mortality for mothers and newborn babies despite many efforts to improve access and quality to healthcare. A key problem is that there is very little accurate data on the resources and facilities available at health centres in the country, particularly in more rural and remote areas.

We visited all 63 health facilities within one health district, Vanga, located within Kwilu province, South-Western DRC. At each facility, we collected data to assess the quality of healthcare in several areas, including case registers from two three-month periods, infrastructure, staff, equipment, supplies and medicines, and key emergency obstetric and neonatal services offered.

The data showed widespread shortages of the most basic infrastructure, such as beds, toilets and running water. Facilities were greatly short-staffed and not properly trained to handle the cases they were performing. Facilities lacked the most basic supplies and medicines. Some facilities did not provide core emergency services such as delivering antibiotics to pregnant and post-parum women, whereas others reported performing caesarian sections and blood transfusions without adequate training or doctors. There was evidence that cases being recorded in registers were falsified for financial incentives, which cast doubt on the validity of the caseload data.

These findings underscore the failings of obstetric and neonatal care provided in rural areas of the DRC, and suggest patients are being harmed. Policymakers and researchers should work closely with local health managers to identify and implement strategies to improve the validity of caseload data and address critical shortages in resource allocation.

## 2 Introduction

Reducing maternal and neonatal death in sub-Saharan Africa is a global health priority. Rates of mortality in sub-Saharan Africa remain unacceptably high: neonatal mortality rate (NMR) in the DRC in 2023 was estimated to be 25.3 per 1,000 births ^1^ - over twice the UN Sustainable Development Goal target (UN SDG) of ≤ 12 deaths per 1000 livebirths by 2030 ^2^. Maternal mortality rate in the DRC in 2022 was estimated at 620 per 100,000 live births ^3^ - nearly ten times the UN SDG target of <70 per 100,000 by 2030 ^4^. While a large body of research has focussed on improving access to healthcare, the quality of care in extremely low-resource settings is a persistent challenge that risks being overlooked as attention turns to development, testing and scaling up new technologies ^5^.

To improve the quality of emergency obstetric and neonatal care (EmONC), existing care provision must be regularly assessed with deficits recognised and then addressed. This requires a means of assessing quality within healthcare facilities in Sub-Saharan Africa as a multi-dimensional construct, encompassing quantity and skill-level of human resources, equipment, infrastructure, organisation and recorded patient outcomes. There are several validated data collection tools designed to enable standardised facility assessment, including the EmONC framework ^6^, the United Nations’ Population Fund Service Provision Assessment (UNPF SPA) ^7^ and the World Health Organisation’s Service Availability and Readiness Assessment (WHO SARA) ^8^. The EmONC signal functions are a set of nine clinical tracer interventions—seven basic (BEmONC) and two comprehensive (CEmONC)—representing practices which evidence shows have the greatest impact in mitigating maternal and newborn morbidity and mortality. We utilised adapted survey tools based on these standardised approaches and built on the Donabedian model of assessing the quality of healthcare systems: evaluating structure, process and outcome ^9^. This report specifically assesses the quality of emergency obstetric and neonatal care (EmONC) provided within all 63 healthcare facilities (excluding the general referral hospital) in Vanga district, Kwilu province, DRC. Vanga district, in the western/central area of DRC is taken as an example of a rural district likely to indicate which challenges still undermine the provision of essential elements of a functioning primary health care system that should support maternal and neonatal health in a low-resource rural setting.

## 3 Methods

### 3.1 Context

The DRC contains 26 provinces divided into 516 health zones (health districts). Each health zone contains a general referral hospital and multiple smaller health areas served by a single health centre.This study was carried out in the Vanga health zone, Kwilu province, where one lead author is a practicing doctor with understanding of the healthcare network, local geography, language and customs, enabling successful access to facilities and available data. Vanga, east of Kinshasa, is bordered by four health zones: Djuma to the north and east, Bulungu to the south, and Bonga Yasa and Musangu to the west. Vanga faces substantial infrastructural challenges with no functioning roads in many areas. Transportation relies on bicycles and motorbikes as other means are largely unavailable. Healthcare facilities cover an area of approximately ~2,600km ^2^ for a population of ~362,465 that is mostly rural and economically impoverished ^10^. Small-scale farming is the main livelihood. Pregnant women represent 4% of the population ^10^. The Vanga health zone is reported as containing 65 health facilities and one general referral hospital serving 43 health areas, the remotest of which is 90 km from the central town and first referral hospital in Vanga. However, two health facilities were found to be nonexistant, leaving 63 health facilities and one hospital serving 119 villages.

The DRC has published guidelines for the provision of mother and newborn care. These define health facility requirements in the domains of human resources, infrastructure, materials, medicines and other tools, to provide quality care, see Table 1. These guidelines divide health facilities as “lower level” and “higher level” corresponding to the services they are expected to provide (basic or comprehensive EmONC), each with different requirements. However, in reality, facilities in Vanga are not formally categorised. Five health facilities in Vanga employ at least one medical doctor and are expected to deliver the higher level of comprehensive EmONC (to include blood transfusions and caesarian sections) (see Table 2). We group these as “Type A” facilities. 58 health centres did not employ a medical doctor and are expected to deliver basic EmONC (see Table 2), we group these as “Type B” facilities.

**Table 1.** DRC ministry of public health 2012 set of minimum infrastructure, human resource and material requirements for healthcare facilities. See full document in supplementary materials.

	Minimum Requirements for the Delivery of Qualified Assistance at Childbirth as per DRC ministry of public health guidelines
Intervention standard domain	Health centre
**Intervention standards**	12 standards to include use of a partograph, essential care for the newborn and timely referral
**Human resource standards**	Specific personnel standards in include two midwives on duty
**Infrastructure standards**	12 standards to include labour room and treatment room
**Material resource standards**	74 standards to include specific equipment such as a light source and examination table, medications such as antibiotics and antihypertensives and consumables such as sterile gloves and chlorine-based disinfectant

**Table 2.** WHO EmONC signal functions, adapted from WHO MoNITOR 2009 ^16^.

Basic EmONC (bEmONC) signal functions, expected from all facilities	Comprehensive EmONC (cEmONC) signal functions, expected from only general referral hospitals (higher level care centres)
***Perform 1–7 signal functions***	***Perform 1–7 signal functions and 8–9***
(1) Administer parenteral antibiotics	(8) Perform surgery, e.g. caesarean section
(2) Administer uterotonic drugs	(9) Perform blood transfusion
(3) Administer parenteral anticonvulsants for pre-eclampsia and eclampsia	
(4) Manually removal of the placenta	
(5) Removal of retained products of conception	
(6) Perform assisted vaginal delivery	
(7) Perform basic neonatal resuscitation	

Vanga health zone has been part of the World Bank’s performance-based financing program since 2016 ^11^. Part of this program aims to improve healthcare access and quality for mothers and newborns. Under the program, facilities receive payments every three months based on the number and quality of services they provide, focusing on maternal, newborn, child, and reproductive health care. Quality is assessed using a standardised checklist. Two types of contract are used: 1) the minimum package of activities (MPA) for core preventive and curative primary health services and, 2) the complementary package of activities (CPA) for services delivered at first-level referral centres, e.g. complicated deliveries, blood transfusions and surgeries requiring anaesthesia. There are 18 MPA service indicators across maternal and childhood care. Examples include (with reimbursement values converted to $USD): deliveries attended by skilled birth attendants with filled partographs ($5.52) or postnatal consultation between three and seven days post delivery ($1.20) ^11^. There are 22 CPA service indicators, for example: uncomplicated delivery ($9) and caesarian sections ($42). Payments are subject to quality weighting adjustments calculated by standardized tools. Maternity quality indicators are weighted at 12% and include correct use of partogram, availability of supplies, cleanliness and confidentiality ^11^.

Despite World Bank and other financial pay-for-performance initiatives, total health spending in the DRC remains low, estimated at $21 USD per person in 2019 (government spending (16%), external aid (40%), out-of-pocket spending (40%), other (4%)) ^12^, substantially lower than the low-income country average ($34) and the sub-Saharan African average ($85) ^13^.

### 3.2 Study design

***Overview***

We conducted a cross-sectional survey of all Vanga health zone facilities excluding the general referral hospital between September and December 2023. Data in three domains was collected at each facility:

1. Caseload registry data from two periods: Jan-March 2021 and Jan-March 2022
2. Facility infrastructure, human resource and material data was collected using an inventory checklist.
3. Signal function data and type of case handled (in the last twelve months) was collected through a structured questionnaire and registries.

**Figure 1.**
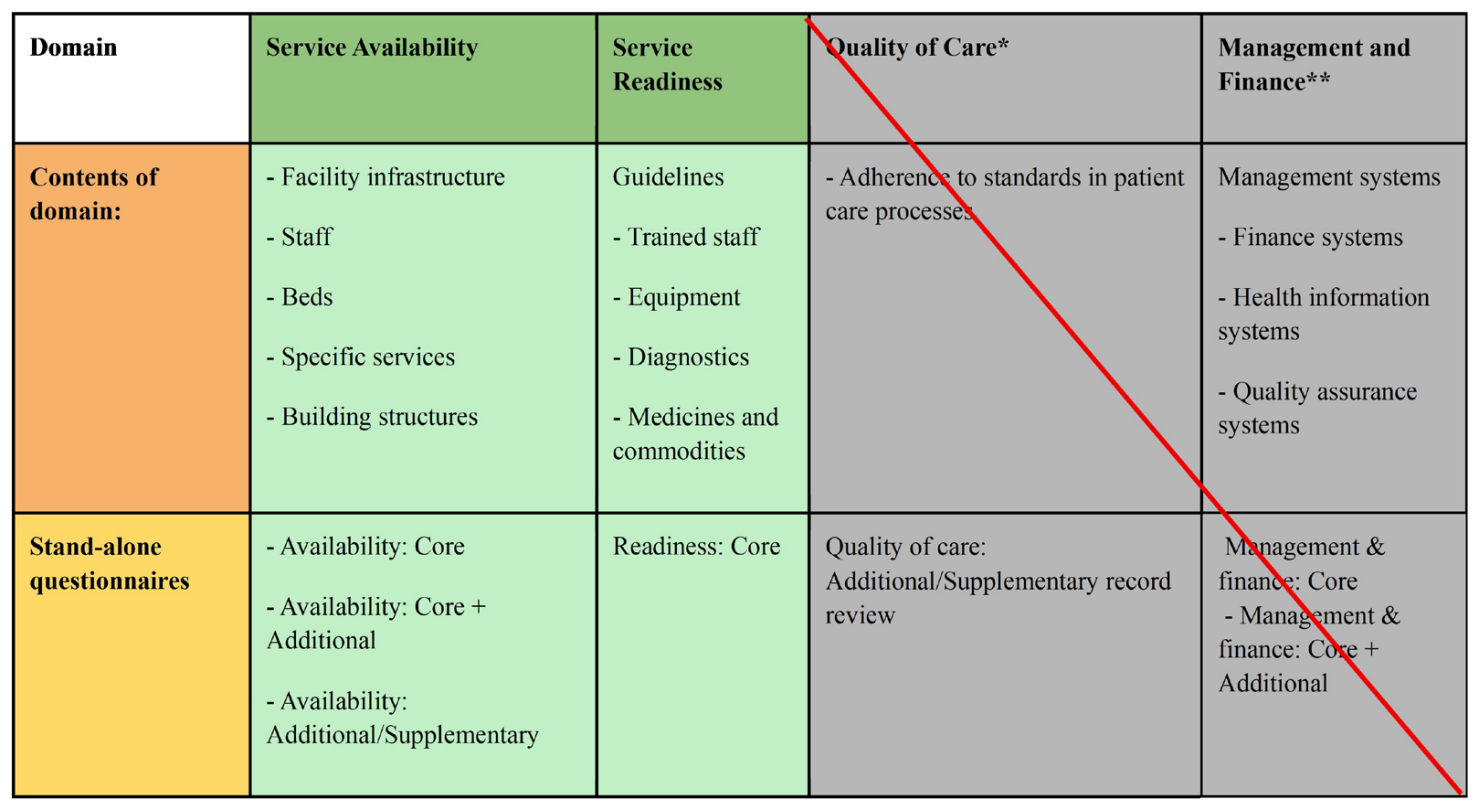
Overview of WHO HHFA modules and questionnaires used by the authors to provide information on the availability and quality of obstetric and neonatal healthcare within the Vanga health zone. Adapted from WHO HHFA Quick Guide 2022 ^11^). *It was not possible to include the quality-of-care domain due to suspicions of falsification of data. **Management and finance were deemed beyond the scope of the paper and is not included.

The survey was based on the WHO 2022 Harmonised Health Facility Assessment tool (HHFA) ^14^ but our survey was more focused in scope and did not extend to examining management and financing. See Figure 1 for a schematic overview of domains assessed.

**1. Caseload data**

We collected caseload data from each facility for two 3-month periods from Jan-March 2021 and Jan-March 2022. Categories of data collected included number of live births, vaginal deliveries, emergency caesarean sections, transfusions, referrals to type A and type B facilities and maternal and neonatal complications.

**2. Facility infrastructure, human resource and materials**

We used a French language facility survey questionnaire developed by Habonimana *et al.* 2022 ^15^. This tool assessed both service availability and readiness based upon the WHO HHFA tool. We referenced the DRC ministry for Public Health guidelines to contextualise the HHFA checklist and focus on basic essential items ( Table 1). We recorded demographics of staff working at each facility.

To qualitatively assess resource availability aggregated across facilities (stratified as Type A, CEmONC, or Type B, BEmONC), we used a grading system adapted from Kosgei 2016 ^17^. For the 5 type A facilities, if 5/5 had the indicator item present, it was graded “Excellent”, 4/5 “Good”, 3/5 “Moderate” and 2 or fewer/5 “Poor”. For the 58 Type B facilities, If >90% facilities had the indicator item present, it was graded '"Excellent”, 89-75% “Good”, 74-50% “Moderate” and <50% “Poor”.

**3 EmONC signal functions**

We recorded EmONC signal functions at each facility. The 9 signal functions are summarised in Table Two. There are seven basic EmONC (BEmONC) signal functions and two comprehensive EmONC (CEmONC) signal functions. Signal functions are identified as the key medical interventions used to treat direct obstetric complications causing the vast majority of deaths worldwide. We assumed type B facilities should perform BEmONC signal functions only while type A facilities should perform all signal functions. Where facilities failed to perform an expected signal function we assessed why.

***Data Collection***

Data collection was conducted by three collaborators familiar with local language, customs and culture working within the Vanga Health zone between September and December 2023 **.** These were: the principal investigator (PI) (consultant physician); a trainee physician and a senior nurse. At each facility, one investigator spent an entire day collecting data. Rigorous steps were taken to ensure consistency in data collection: the PI trained the investigators on how to collect data using a standardized checklist and methodology. Equipment, supplies and medicines were identified and recorded at each facility and, where appropriate, checked to be in working order. Signal functions were assessed by collecting logbooks from the three months prior. To ensure consistency and minimise bias, the PI observed practice rounds whereby investigators were supervised collecting data. The investigators then independently collected data from a sample of facilities in duplicate with the PI so data could be evaluated for inconsistency. Throughout data collection, the PI sampled healthcare facilities to check the accuracy of data collection.

***Data Analysis***

The data were presented in tabular form. Quantitative variables were described using the minimum, 25th percentile, median, 75th percentile, and maximum. Categorical variables were described using absolute frequencies and percentages. Statistical analysis of the data was performed using Stata/BE 18.5 and STATA/BE 19.5 software.

***Research Ethics Approval***

Ethical approval was obtained from the Comité National D'éthique De La Santé (the National Health Ethics Committee) of the Democratic Republic of Congo nº476/CNES/BN/PMMF/2023 on 25/08/2023. All procedures involving human participants were conducted in accordance with the ethical standards of the institutional and/or national research committee and with the 1964 Declaration of Helsinki and its later amendments. Written informed consent was obtained from all participants prior to participation. Participants were informed about the study’s purpose, procedures, potential risks, and confidentiality measures before providing signed consent. No minors were involved in the study. The consent form can be found in supplementary materials.

## 4 Results

### Summary

Below we report key assessment findings for Type A and Type B facilities. Full data are provided in supplementary files A and B. Overall, broad system-level deficiencies were evident, with shortages of trained personnel, essential drugs, equipment, and a lack of both training and adherence to national guidelines and operating standards. EmONC signal function data - the WHO’s minimum standards of obstetric and neonatal care - revealed large deficiencies in the provision of core services. Signal function data also showed several facilities performed CEmONC services such as caesarian sections when not equipped with the trained staff required to provide these services safely, suggesting either that facilities were acting beyond their capability, or that service records are being falsified to access financial incentives (see Section 4.1 below).

Two of the 65 officially listed health centres were nonexistent when attempts were made to visit them and Vanga general referral hospital was not surveyed. We therefore surveyed 63 health centres over eight weeks between September and December 2023; 61/63 (97%) of facilities were public, 2/63 (3%) were private. 5/63 (8%) of facilities employed doctors (Type A facilities), 58/63 (92%) did not (Type B facilities).

### 4.1 Type B facility caseload data from registers

Upon initial analysis of caseload data, we suspected widespread falsification of results. The data from the first quarter of the years 2021 and 2022 was often identical or nearly identical for all service categories within a specific facility, including number of live births and complications. Informal discussion with local service providers suggested a culture falsification of results exists to ensure that figures are in-line with DRC government targets and so that, given major resource challenges, the facility receives regular pay-for-service reimbursement. This rendered caseload data unusable as a form of reliable information.

### 4.2 Type B facility infrastructure, human resource and material

Every health centre 58/58 (100%) reported being open 24hrs a day seven days a week.

***Type B facility basic infrastructure***

Only 2/58 centres (4%) had a reliable water source inside the facility and 16/58 (28%) had working latrines. Zero facilities had internet access though 54/58 facilities (93%) had access to a mobile phone owned by a health worker. 26/58 facilities (45%) used a solar system, the remaining 32/58 (55%) of facilities reported no means of electricity.

No facilities had access to ambulances for emergency transportation or stocked fuel supplies that could support patient transport. No facilities reported availability of blood storage refrigerators but 22/58 (38%) of facilities reported capability of blood screening for blood-borne disease prior to transfusion, suggesting any transfusion performed (a service not included in the BEmONC package) was linked to contemporaneous donation.

***Type B facility bed capacity***

Type B facilities had a median of 3 maternity beds (IQR, 1-4, max 8) and 0 delivery tables (IQR 0-0, max 2). No type B facilities had specific neonatal areas, cots or incubators and none had a functional space for neonatal resuscitation. No facilities had national guidelines or training manuals for essential childbirth care available, and only 1/58 facilities (2%) had national guidelines or protocols for EmONC available in the facility.

***Type B facility human resource***

The bulk of service delivery in type B facilities is performed by nurses. The median number of nurses deployed to a facility and providing all services (including all outpatient care, for 24/7 service cover) was 4 (1-13). The median number of professional midwives at type B facilities was 0 (IQR 0-1, max 2) while the median number of ‘matrons’ (equivalent to a healthcare assistant for maternity care who has not received any formal training) was 1 (IQR 0-1, max 2). All facilities reported routinely deploying at least one nurse/ matron within the facility to offer maternity services including antenatal and delivery care. The median number of vaginal deliveries performed in the last three months was 31 (min 3, IQR 57-15, max 95).

Almost no facilities had access to trained laboratory technicians (median 0, IQR 0-0 max 1). Not all facilities employed cleaning staff (median 1 IQR 1-2, min 0, max 3).

We also assessed the qualification level and demographics of nursing staff. 81% of nurses were female (19% were male). The median age of nurses varied. See supplementary file B. All nurses and midwives reported at least one year of clinical experience (IQR 1-21, median: 9) and all had performed at least one delivery in the past three months (IQR 15-60, median: 32). The median years of experience service providers reported since completing their most recent professional qualification was 9 years (min 1, IQR 20-5, max 29).

***Staff training at Type B facilities***

At any given facility, the maximum number of maternity service providers who had received training was 1. Only 11/58 facilities (19%) reported receiving EmONC training. All 11 facilities that had reported EmONC training had received training within the past two years.

In 34/58 type B facilities (59%), service providers did however, report receiving some continuing education in maternal and neonatal health (consisting of family planning and antenatal care) since starting their employment. Only 8/58 (14%) type B facilities reported receiving continuing education in BEmONC. Zero facilities reported receiving continuing education in CEmONC.

***Type B availability of equipment, supplies and medicine***

**Maternal and obstetric equipment**

The data revealed stark deficiencies in the availability of equipment, supplies and medications across all facilities. Using the availability grading system adapted from Kosgei 2016 ^17^ (available in >90% facilities, Excellent; in 89-75%: Good; in 74–50%: Moderate and in less than 50%: Poor) the data shows poor overall availability of obstetric and maternal equipment. Only 3 of 21 items (two pairs of sterile gloves, two gynaecological disinfectants and a scissor/blade for cutting umbilical cord) were assessed as being “good” on the availability grading system. One item, blank partogram, was assessed “Moderate”, present in 31/58 (54%) facilities. 18 of the 21 items (86%) were assessed “Poor”. Across items assessed “Poor”, the median number of facilities stocking a maternal or obstetric item was 2/58 (4%) ( Table 3).

**Table 3.** Equipment, supplies and medications found in less than 50% of Type B facilities.

Indicator items classified poor (Found in <50% Type B facilities) by domain
Obstetric and maternal equipment (n = 23)
Cord clamp 10/58 (17%) episiotomy scissors 12/58 (21%) Suture material with needle 24/58 (41%) needle holder 13/58 (22%) 2 sterile drapes 70 x 70 2/58 (4%) 2 x 5 sterile compresses 7.5 x 7.5 13/58 (21%) 2 3-ply masks 4/58 (7%) 2 jackets 4/58 (7%) 1 sterile newborn woad cannula t 000 1/58 (2%) 1 sterile pediatric suction probe ch 6 2/58 (4%) 1 biconical connector 2/58 (4%) 2 sterile umbilical clamps 1/58 (2%) 1 cap 2/58 (4%) 1 sterile isothermal sheet 1/58 (2%) 1 high concentration adult O2 inhalation mask 1/58 (2%) 1 very high concentration pediatric O2 inhalation mask 1/58 (2%) 1 pair of sterile scissors 11/58 (19%) Examination lamp 2/58 (4%) Anesthesia machine for delivering aesthetic gases and oxygen 0/58 (0%)
Specialist neonatal care equipment (n = 16)
Intubation Kit - Pediatric (complete with oropharyngeal airway, endotracheal tubes, laryngoscope, Magill forceps , stylet) 0/58 (0%) Suction cup / forceps 0/58 (0%) Fetal monitoring device 0/58 (0%) Ultrasound scanner 0/58 (0%) Pulse oximeter – pediatric 0/58 (0%) Pulse oximeter – neonatal 0/58 (0%) Phototherapy unit 0/58 (0%) Operating table 1/58 (2%) Baby scale in the delivery room 23/58 (40%) Blood pressure measuring device in the delivery room 10/58 (17%) Suction device (suction bulb or electric suction pump) 2/58 (4%) Neonatal bag and mask size 1 - for full-term babies 0/58 (0%) Neonatal bag and mask size 0 - for premature babies 0/58 (0%) Resuscitation table with heat source for newborn resuscitation 1/58 (2%)
Medications (n = 11)
Calcium gluconate 0/58 (0%) Hydralazine 0/58 (0%) Antibiotic eye ointment for newborns 0/58 (0%) Azithromycin (cap/tablet or oral liquid) 0/58 (0%) Magnesium sulfate 0/58 (0%) Misoprotol 1/58 (2%)

**Neonatal equipment**

Availability of specialist neonatal equipment was also poor. Only 1 of 15 items (sterile gloves) was assessed “Good”, available in 47/58, 81% of Type B facilities; mirroring good availability of gloves for obstetric care. All other items, 14 of 15 (93%) were assessed “Poor”. The median number of facilities stocking a given neonatal item was 1/58 (2%). 8/15 neonatal care items (47%), were not stocked by a single facility ( Table 3).

**Medications**

Availability of medications was improved compared to other categories. Availability of 2/11 items were assessed as “Excellent”, gentamicin 56/58 (97%) and metronidazole 54/58 (93%). Ampicillin was assessed “Good” 45/58 (78%) while oxytocin 42/58 (72%) and sodium chloride (saline) solution 43/58 (74%) were both assessed “moderate”. Six of 11 items (55%) were assessed as “poor”. The median number of facilities stocking a given medication was 14/58 (24%). No type B facilities stocked calcium gluconate or hydralazine ( Table 3).

### 4.3 Type B facility EmONC signal functions

We collected data on the WHO’s EmONC signal functions across health facilities. Where facilities reported not performing a signal function we assessed reasons why. Findings are summarised in Table 4. The most commonly performed signal function was administration of parenteral uterotonics (54/58, 93%). Removal of retained products of conception was also performed by a large proportion of facilities (47/58, 81%). Just under half (28/58, 48%) reported administering parenteral antibiotics while a third (19/58, 33%) performed manual placental extraction. No facilities performed assisted delivery or neonatal resuscitation and only 1/58 facilities administered anticonvulsants for pre-eclampsia or eclampsia. The most widely cited reason for signal functions not being performed were supply chain and equipment issues and inadequate training.

**Table 4.** Type A and B facility EmONC signal functions.

Signal function	Have parenteral antibiotics been administered to a pregnant or recently delivered woman within the past 3 months? (%)	Have any parenteral uterotonics been administered within the last 3 months? (e.g., parenteral oxytocin, ergometrine, misoprostol) (%)	Have anticonvulsants for severe preeclampsia or eclampsia been administered parenterally within the past 3 months? (e.g., magnesium sulfate) (%)	Has manual placental extraction been performed within the last 3 months? (%)	Has removal of retained products of conception been performed within the last 3 months? (e.g., manual vacuum aspiration, dilation and curettage) (%)	Has an assisted vaginal delivery (e.g., vacuum extraction, forceps delivery) been performed within the last 3 months? (%)	Has neonatal resuscitation with a bag and mask been performed in the last 3 months? (%)	Has a blood transfusion been performed in the last 3 months? (%)	Has a cesarean section been performed in the last 3 months? (%)
Type A facilities									
**Done within the last 3 months? (Yes)**	5/5 (100)	5/5 (100)	0/5 (0)	2/5 (40)	5/5 (100)	0/5 (0)	1/5 (20)	5/5 (100)	5/5 (100)
**Not done in the last three months (No)**	0/5 (0)	0/5 (0)	5/5 (100)	3/5 (60)	0/0 (0)	5/5 (100)	4/5 (80)	0/5 (0)	0/5 (0)
**If not done in the last three months, why?**									
**Training issues**	0 (0)	0 (0)	0 (0)	2 (40)	0 (0)	0 (0)	0 (0)	0 (0)	0 (0)
**Supplies, equipment, medication issues**	0 (0)	0 (0)	1 (20)	0 (0)	0 (0)	5 (100)	4 (80)	0 (0)	0 (0)
**Management problems**	0 (0)	0 (0)	0 (0)	0 (0)	0 (0)	0 (0)	0 (0)	0 (0)	0 (0)
**Policy issues**	0 (0)	0 (0)	0 (0)	0 (0)	0 (0)	0 (0)	0 (0)	0 (0)	0 (0)
**No indication**	0 (0)	0 (0)	4 (80)	4 (80)	0 (0)	0 (0)	0 (0)	0 (0)	0 (0)
**Type B facilities**									
**Done within the last 3 months (Yes)**	28/58 (48)	54/58 (93)	1/58 (2)	19/58 (33)	47/58 (81)	0/58 (0)	0/58 (0)	19/58 (33)	7/58 (12)
**Not done in the last 3 months (No)**	30/58 (52)	4/58 (7)	57/58 (98)	39/58 (67)	11/58 (19)	58/58 (100)	58/58 (100)	39/58 (67)	51/58 (88)
**If not done in the last three months, why not?**									
**Training problems**	3/30 (10)	2/4 (50)	14/57 (25)	7/39 (18)	17/58 (36)	24/58 (41)	40/58 (69)	17/39 (44)	7/51 (14)
**Supplies, equipment, medication issues**	19/30 (63)	4/4 (100)	36/57 (63)	7/39 (18)	27/58 (57)	53/58 (91)	50/58 (86)	27/39 (69)	6/51 (12)
**Management problems**	1/30 (3)	1/4 (25)	1/57 (2)	0/39 (0)	0/58 (0)	0/58 (0)	1/58 (2)	1/39 (3)	3/51 (6)
**Policy issues**	3/30 (10)	1/4 (25)	17/57 (30)	2/39 (5)	12/58 (26)	5/58 (9)	4/58 (7)	27/39 (69)	1/51 (2)
**No indication**	10/30 (33)	2/4 (50)	37/57 (65)	34/39 (87)	27/58 (57)	6/58 (10)	9/58 (16)	4/39 (10)	48/51 (94)

Despite not having medical doctors, a third (19/58) of type B facilities reported performing blood transfusions, conducted without laboratory professionals’ support for cross-matching of products or proper blood product refrigeration capacity. Only 2/19 Type B facilities performing transfusions reported no interruptions in the availability of blood products in the last 3 months. Concerningly, 7/58 Type B facilities (12%) reported performing caesarean sections though they were only staffed by nurses. These nurses have not received formal training in performing caesarian sections, instead obtaining experience from shadowing colleagues. These nurses are also responsible for use of the anaesthetic (ketamine), again without formal training.

### 4.4 Type A facility caseload data

***Type A caseload data***

Due to the falsification problem outlined in Section 4.1, a decision was made not to include caseload data, through lack of credibility in the results.

### 4.5 Type A facility infrastructure, human resource and materials

Type A facilities are defined as those employing at least one medical doctor. Vanga referral centre was not included in the study.

***Type A facility basic infrastructure***

One of five (20%) type A facilities had access to a reliable water source powered by solar energy. Two of five (40%) had internet access. Five of five (100%) facilities had access to a private mobile phone. No type A facility had access to ambulances for emergency transportation or fuel supplies. All five (100%) had electricity supply via solar systems. All facilities (100%) reported being open 24hrs a day 7 days a week. Four of five facilities (80%) reported the capacity to screen blood products.

***Type A facility bed capacity and environment***

Median total bed capacity was 22 (16, 19, 22, 22, 45), median maternity bed capacity was 7 (4, 7, 7, 9). 2 of 5 facilities had specific delivery beds. Despite performing caesarian sections, no type A facilities had operating rooms or surgical beds. Caesarean sections were performed on patient gurneys. No type A facilities had beds for newborn care, nor did any facility (type A or B) provide basic neonatal care such as kangaroo mother care for premature/low birthweight babies or skin-to-skin contact support. No type A facilities had specific neonatal areas, beds or incubators and none had a functional space for neonatal resuscitation. No type A facilities had national guidelines, manuals or protocols for EmONC available on site.

***Type A facility human resource***

Four of five type A facilities had 12 nurses and one had 15 nurses (median 12). These staff provide all services including outpatient care for 24/7 care. The median age of nurses varied across facilities (see supplementary materials). Four of five facilities (80%) employed a single midwife. Two facilities (40%) employed 2 matrons (healthcare assistants who have not received formal training), one facility (20%) employed one matron and two facilities (40%) had zero matrons. No gynaecologists, paediatricians or neonatologists work in the Vanga healthzone. GPs (defined as medical graduates with no further postgraduate training) were the only medical specialty available, and only at the five Type A facilities. Two facilities had 2 GPs, while three facilities each had 3 GPs. These doctors served the entire facility and were not dedicated to maternity care.

***Type A availability of equipment, supplies and medicine***

Availability of equipment, supplies and medicine across type A facilities was poor. We classified availability of items in 5/5 facilities as “Excellent”; in 4/5 as “Good”; in 3/5 as “moderate” and 2 or fewer out of 5 as “Poor”.

***Obstetric and maternal equipment***

The availability of obstetric and maternal equipment was inadequate. Only 5 of 23 (22%) items (two pairs of sterile gloves, sterile scissors, sterile compresses, blank partogram and disinfectant) were assessed “Excellent”. Availability of all other items in this category was “Poor” (see Table 5). 2/23 (9%) maternal and obstetric items (sterile isothermal sheet, adult high concentration O2 mask) were stocked by 0 facilities.

**Table 5.** Equipment, supplies and medications found in less than 50% of Type A facilities.

Indicator items classified as poor (Found in <50% Type A facilities) by domain
Obstetric and maternal equipment (n = 23) (%)
Examination lamp 1/5 (20) Suction device 1/5 (20) Epistiotomy scissors 1/5 (20) Needle holder 2/5 (40) 2x Sterile drapes 1/5 (20) 2x Sterile 3-ply masks 1/5 (20) 2x sterile jackets 1/5 (20) 1 cap 1/5 (20) 1 sterile isothermal sheet 0/5 (0) high concentration adult O2 inhalation mask 0/5 (0) Blank partogram 1/5 (20) Operating table 1/5 (20)
Specialist neonatal care equipment (n = 18) (%)
1 sterile newborn woad cannula t 000 1/5 (20) 1 sterile pediatric suction probe ch 6 1/5 (20) 1 biconical connector 1/5 (20) Anesthesia machine for delivering aesthetic gases and oxygen 0/5 (0) Intubation Kit - Pediatric (complete with oropharyngeal airway, endotracheal tubes, laryngoscope, Magill forceps , stylet) 0/5 (0) 1 very high concentration pediatric O2 inhalation mask 1/5 (20) 2 sterile umbilical clamps 0/5 (0) Suction cup / forceps 0/5 (0) Fetal monitoring device 0/5 (0) Ultrasound scanner 0/5 (0) Pulse oximeter – pediatric 0/5 (0) Pulse oximeter – neonatal 0/5 (0) Phototherapy unit 0/5 (0) Baby scale in the delivery room 2/5 (40) Blood pressure measuring device in the delivery room 2/5 (40) Suction device (suction bulb or electric suction pump) 1/5 (20) Neonatal bag and mask size 1 - for full-term babies 0/5 (0) Neonatal bag and mask size 0 - for premature babies 0/5 (0) Resuscitation table with heat source for newborn resuscitation 0/5 (0)
Medications (n = 11)
Calcium gluconate 0/5 (0) Hydralazine 0/5 (0) Antibiotic eye ointment for newborns 0/5 (0) Azithromycin (cap/tablet or oral liquid) 0/5 (0) Magnesium sulfate 0/5 (0) Misoprotol 2/5 (40) Sodium chloride solution (injection) 1/5 (20)

***Specialist neonatal equipment***

Availability of specialist neonatal equipment was very poor. Only 2 of 18 items (11%) (sterile gloves, and baby weighing scales) were assessed “Excellent”. All other neonatal items, 14 of 18 (78%) were assessed “Poor”. 12/18 neonatal care items (67%) were not available in any facility.

***Medications***

Availability of medications was improved compared with other categories. Three items were assessed “Excellent” (gentamicin, metronidazole and ampicillin) stocked by all five facilities. Oxytocin (4/5 80%) was assessed “Good”. Six of 11 items (55%) were assessed “Poor”. No type B facilities stocked calcium gluconate, hydralazine or magnesium sulfate (3/11 27%).

***Type A facility staff training and experience***

Four of five type (80%) Type A facilities had received BEmONC training. Zero facilities had received CEmONC training. Four of 5 (80%) facilities also received continuing education in maternal and neonatal health to include family planning and antenatal care.

### 4.6 Type A facility EmONC signal functions

EmONC signal function data collected from Type A facilities is summarised in Table 4. All Type A facilities reported performing the BEmONC signal functions of administering parenteral antibiotics, uterotonics and removal of retained products of conception. However, none reported administering anticonvulsants or performing assisted vaginal delivery and only two and one Type A facility reported performing manual removal of the placenta and neonatal resuscitation respectively. Conversely all five had performed both blood transfusion and caesarian section, the CEmONC signal functions, in the previous 3 months.

## 5 Discussion

### Summary

We surveyed the 63 local health facilities operating within Vanga healthzone (excluding Vanga general referral hospital), Kwilu province, DRC to assess the quality of care provided to mothers and newborns. While ‘on paper’, the population served by the Vanga health zone may appear to have good access to maternal and neonatal care (63 health facilities and 1 general referral hospital for a population of approximately ~362,465) ^10^, our new data suggests this to be misleading due to severe inadequacies across all domains assessed. These results reveal that the current levels of infrastructure, human resource, equipment, supplies, medicines do not display a readiness to provide BEmONC, or in Type A facilities, CEmONC as outlined by the WHO or local guidelines.

Furthermore, our data raises concerns over widespread implementation of a number of dangerous practices that may be causing harm to patients, including unsafe blood transfusions (in both type A and B facilities) and caesarean sections performed in the absence of doctors, equipment or operating theatres.

Our study has also raised questions over the effectiveness and possible harms of pay-for-service schemes through incentivisation of falsification of results.

### Infrastructure, availability of equipment, supplies and medicine

The lack of availability of basic infrastructure and resources suggests obstetric and neonatal care within the Vanga healthzone is dangerously undersupported and underequipped, raising serious concerns of unsafe practice and patient harm. Almost all (61/63, 95%) of health facilities have no source of water, more than half have no electricity and those that do rely on solar power. There was no capacity to transfer patients, rendering access to higher care non-existent. Only 2/63 facilities had internet access only and 59/63 (94%) facilities had access to a mobile phone, meaning access to emergency consultation or guidelines is virtually non-existent. There is also direct evidence of unsafe practice. Zero type B facilities reported blood refrigeration capacity, yet 19/58 report performing transfusions. The practice of such contemporaneous transfusions outside of the cold-chain risks the viability of blood products and greater infection risk, especially due to the absence of trained laboratory staff, suggesting that adequate cross-matching is not taking place.

Bed capacity was inadequate, with near-zero delivery tables (median 0, IQR 0-0). This suggests that deliveries and in some cases, caesarian sections, were being carried out in beds not clinically fit for purpose.

### Human resource concerns

Our results revealed major human resource constraints on service delivery in the Vanga health zone. Service delivery is reliant upon nurses, and there was at least one nurse at each health facility (median 6 (min 1- max 15) nurses at type A facilities, median 2 (min 1- max 4) at type B facilities). There were few midwives and the presence of doctors was rare, limited to 13 physicians operating across 5 health centres. There were zero specialists working in the health zone, with all physicians being ‘GPs’ - defined as medical graduates with no postgraduate training. These figures represent the total staff numbers for health centres and, as all 63 facilities purport to offer 24/7 care, the data suggests staff are working vastly extended hours, or facilities are in fact not offering a full range of services/hours. In centres employing 1–2 nurses, where sickness/absence occurs, service delivery is presumably constrained.

There were several major patient safety concerns raised by the data collected. Human resource data revealed widespread evidence of staff acting beyond their official competence or capability. For example, 7/58 type B facilities report performing caesarian sections and 18/58 type B facilities report performing blood transfusions, both without any doctors present. A possible explanation for the high rates of unsafe interventions being performed is the lack of alternative options: zero facilities had access to ambulances to transfer patients. This evidence suggests it is highly likely that harm is being done to patients through system-level failings in staff and infrastructure resourcing.

The WHO has warned that there is a global shortage of health workers, with a need for 900 000 more midwives worldwide ^18^. It is estimated that fully resourcing midwife-led care could reduce maternal mortality by 67%, newborn mortality by 64% and stillbirths by 65% ^19^. The DRC faces a major shortage of doctors, with an estimated 0.35 medical doctors per 10,000 inhabitants ^20^. Our results illustrate the consequences of workforce shortages likely to be compounded by difficulties with recruitment and retention due to geographical isolation, insufficient support and a fundamental lack of basic infrastructure, which are likely to disincentivize staff to remain. The DRC’s ability to improve its recruitment and allocation of skilled healthcare staff while it addresses basic infrastructure and resource constraints will be central to any effort to improve its obstetric and neonatal patient outcomes.

### EMONC signal functions and safety concerns

There is a lack of training for healthcare staff in the provision of EmONC and a large number of unsafe practices. For example, less than 30% of healthcare staff involved in childbirth have received formal training in emergency obstetric and neonatal care. Perhaps as a result and reflecting absence of equipment, only 5% of facilities reported offering assisted vaginal delivery and very few had experience or capacity to administer anticonvulsants to women with pre-eclampsia / eclampsia. In contrast, blood transfusion was reported in more facilities than one might expect, with the majority of those performing blood transfusion not able to screen for infectious diseases or even perform cross-matching prior to transfusion.

There are particular concerns over readiness to offer essential forms of neonatal care. No facilities had a dedicated space and equipment for resuscitating newborn babies. Only one of 63 facilities (a Type A facility) reported performing neonatal resuscitation, and equipment or medicines for providing this or other forms of newborn care were almost universally non-existent in health facilities.

### Comparison with existing literature

Our study echoes previous research identifying systemic deficiencies in provision of EmONC services in the DRC. In a large facility-level cross-sectional study of 42 facilities in 3 provinces in DRC, Mizerero 2021 ^21^ identified that the vast majority of facilities did not meet basic EmONC standards. Casey 2015 ^22^ and Casey 2009 ^23^ and of Ntambue 2011 ^24^ also establish failings in DRC facilities to provide adequate EmONC suggesting little progress has been made in addressing the issue. Our results also raise questions over pay for performance (P4P) schemes in the DRC. While evidence suggests such schemes may improve antenatal care in the DRC ^25^, evidence on benefits to neonatal and maternal health outcomes is less convincing ^25^. Our study suggests P4P is conceivably being abused through falsification of patient records to achieve financial rewards. This is especially damaging given the already critical shortage of reliable health data from health facilities within the DRC. These findings echo a growing body of evidence raising questions over such incentive schemes ^15^.

### Limitations

We outline several study limitations. Due to concerns over the credibility of the caseload data, we cannot relate facility survey data on resources and care provision to facility caseload data. Consequently, we cannot link shortages in specific areas to patient outcomes. The cross-sectional nature of the study means we are unable to follow trends in facility services and resources over time. The study included all health facilities (excluding Vanga referral hospital) within the Vanga health zone, a single district in Kilwu province. This limits geographical generalisability of conclusions to the wider DRC. Despite these limitations, we provide evidence of major patient safety concerns and comprehensive data on current infrastructure and operational status of healthcare facilities within the Vanga health zone, DRC. This previously unknown information should be used to inform the challenging process of decision-making on allocation of material and human resources, the need for health worker training, and investments in basic infrastructure in the region.

### Future directions

There is a need for closer collaboration between researchers, district health managers and funding bodies to develop approaches that improve the validity of caseload data and minimise falsification. Alternative approaches to facility financing and their effects on maternal and neonatal outcomes should also be examined given the concerns over P4P schemes in use in rural DRC. As more attention turns to the development of broader integrated primary care systems that include EmONC, researchers should explore methods of supporting supervision, training and local improvement efforts. Likely through mobilising local actors in district offices and local hospitals.

## Conclusion

There is evidence of major deficiency in the quality of EmONC services provided to mothers and babies in the Vanga health zone, Kwilu province, DRC, in every domain assessed. Facilities lack basic infrastructure and are not resourced with the materials or staff to provide adequate EmONC. Many facilities do not perform basic EmONC services, while others perform comprehensive EmONC services such as caesarian sections, blood transfusions and anaesthesia without appropriate staff, training or equipment present. Caseload data is invalid due to suspicions of falsification of records for financial incentives. Taken together, we present evidence that women and babies in the Vanga healthzone are likely suffering avoidable morbidity and mortality through insufficient access to EmONC, and potential harm from dangerous practices.

## Ethics and consent

Ethics approval was received from the Comité National D'éthique De La Santé (the National Health Ethics Committee) of the Democratic Republic of Congo nº476/CNES/BN/PMMF/2023 on 25/08/2023. Written informed consent was obtained from all participants prior to participation.

## Data Availability

The full dataset contains information of a potentially sensitive nature, including evidence of deviation from national guidelines and irregularities in recordkeeping. Due to the small number of facilities within a clearly defined region where data was collected from, there is a real risk of indirect identification of facilities or staff. Ethical approval for the study was granted on the condition that individual-level data would not be made publicly accessible, and for this reason, the full dataset cannot be made freely available through an open-access repository. However, data may be shared upon request by contacting lead author Dr. Junior Mudji, mudjijunior@gmail.com, in accordance with ethical and confidentiality requirements. Figshare. Facility-Based Assessment of Emergency Obstetric and Neonatal Care in Vanga Health Zone, Kwilu Province, Democratic Republic of Congo https://doi.org/10.6084/m9.figshare.30712898 ^26^ This project contains the following underlying data: Figures and Tables. (A full set of included figures and tables). Study Proposal. (A copy of the original study proposal). Supplementary file A. (A full set of data collected from Type A facilities. Supplementary file B. (A full set of data collected from Type B facilities. Data is available under the terms of the CC BY 4.0.
