# Facility-Based Assessment of Emergency Obstetric and Neonatal Care in Vanga Health Zone, Kwilu Province, Democratic Republic of Congo

**DOI:** 10.12688/wellcomeopenres.25137.2

**Published:** 2026-04-27

**Authors:** Mudji Junior, Kieran Desmond, Bill Sabwa, Mike English

**Affiliations:** 1 University of Oxford Nuffield Department of Medicine, Oxford, England, UK; 2 Health Services Unit, Health Services Unit, KEMRI-Wellcome Research Programme, Nairobi, Kenya, Nairobi, Nairobi, Kenya; 3 Institut National de Recherche Biomédicale, Kinshasa, Democratic Republic of the Congo

**Keywords:** Emergency Obstetric and Neonatal Care (EmONC), Maternal Health, Neonatal health, Health facility assessment, Quality of Care, Health system capacity, Democratic Republic of Congo, Resource allocation

## Abstract

**Background:**

Maternal and neonatal morbidity and mortality rates in the Democratic Republic of Congo (DRC) remain unacceptably high. The lack of empirical evidence on the capacity and performance of health facilities offering emergency obstetric and neonatal care (EmONC) is a central problem.

**Methods:**

This study assessed the quality of EmONC provision across 63 healthcare facilities in Vanga Health Zone, Kwilu province, DRC.

**Results:**

We identified widespread infrastructural deficiencies, including no water sources in 61/63 facilities, no emergency transfer capability, and critically low bed capacity. Stock inventories showed that 38/52 categories assessed in facilities had poor availability of basic EmONC equipment, supplies and medications. The median number of nurses providing 24/7 care across all specialties in nurse-led facilities was four. Doctors were employed at 5/63 facilities (13 doctors total), none with postgraduate training. Signal function data revealed widespread failure to provide basic EmONC alongside dangerous practices including caesarean sections and blood transfusions performed without doctors, trained staff or essential equipment. Caseload data was near-identical across both collection periods in all 58 nurse-led facilities, possibly suggesting falsification linked to performance-based financing.

**Conclusion:**

Health facilities in Vanga Health Zone show inadequacies in all quality domains assessed and are unable to provide safe or acceptable EmONC. Women and newborns are suffering unnecessary harm due to system-level failings in resource allocation and governance.

## Plain Language summary

Facility-based Assessment of Emergency Obstetric and Neonatal Care in Vanga Health Zone, Kwilu Province, Democratic Republic of Congo.

The DRC has one of the highest rates of mortality for mothers and newborn babies despite many efforts to improve access and quality to healthcare. A key problem is that there is very little accurate data on the resources and facilities available at health centres in the country, particularly in more rural and remote areas.

We visited all 63 health facilities within one health district, Vanga, located within Kwilu province, South-Western DRC. At each facility, we collected data to assess the quality of healthcare in several areas, including case registers from two three-month periods, infrastructure, staff, equipment, supplies and medicines, and key emergency obstetric and neonatal services offered.

The data showed widespread shortages of the most basic infrastructure, such as beds, toilets and running water. Facilities were greatly short-staffed and not properly trained to handle the cases they were performing. Facilities lacked the most basic supplies and medicines. Some facilities did not provide core emergency services such as delivering antibiotics to pregnant and post-parum women, whereas others reported performing caesarian sections and blood transfusions without adequate training or doctors. There was evidence that cases being recorded in registers were falsified for financial incentives, which cast doubt on the validity of the caseload data.

These findings underscore the failings of obstetric and neonatal care provided in rural areas of the DRC, and suggest patients are being harmed. Policymakers and researchers should work closely with local health managers to identify and implement strategies to improve the validity of caseload data and address critical shortages in resource allocation.

## 1. Introduction

Reducing maternal and neonatal death in sub-Saharan Africa is a global health priority. Rates of mortality in sub-Saharan Africa remain unacceptably high: neonatal mortality rate (NMR) in the DRC in 2023 was estimated to be 25.3 per 1,000 births ^1^ - over twice the UN Sustainable Development Goal target (UN SDG) of ≤12 deaths per 1000 livebirths by 2030. ^2^ Maternal mortality rate in the DRC in 2022 was estimated at 620 per 100,000 live births. ^3^ - nearly ten times the UN SDG target of <70 per 100,000 by 2030 ^4^ While a large body of research has focussed on improving access to healthcare, the quality of care in extremely low-resource settings is a persistent challenge that risks being overlooked as attention turns to development, testing and scaling up new technologies. ^5^

This study is set in the Vanga health zone, Kwilu province, south-western DRC — a rural district representative of the challenges facing much of rural DRC. Vanga serves a population of approximately 362,465 across ~2,600 km ^2^, where small-scale farming is the primary livelihood and economic impoverishment is widespread. ^10^ The zone faces severe infrastructural challenges: roads are largely non-functional, with transportation relying on bicycles and motorbikes, and the most remote health area lies 90 km from the nearest referral hospital. While Vanga is a single district, these characteristics — geographic isolation, poor infrastructure, economic deprivation and limited healthcare access — are common to much of rural DRC, making findings from this setting likely to reflect broader national challenges in delivering maternal and neonatal care outside of urban centres.

An optimal obstetric and neonatal healthcare system can deliver high-quality care in community centres as well as refer complex cases to referral facilities. The EmONC framework defines the minimum standards of care in each type of facility: basic EmONC facilities need to be capable of administering antibiotics, anti-convulsants and uterotonics, manually remove the placenta, retained products of conception, assist vaginal delivery and perform basic neonatal resuscitation. Referral facilities must be able to provide comprehensive EmONC including caesarean sections and blood transfusions. To achieve these standards, each facility needs to meet specific inputs: adequate numbers of skilled staff, a supply of essential medicines, adequate bed space and equipment, and basic infrastructure to provide sanitation, electricity and water. Evidence shows that when these input requirements are met, obstetric and neonatal outcomes improve. ^18^

The DRC health system is organised into 26 provinces subdivided into 516 health zones, each containing a general referral hospital and multiple smaller health centres expected to deliver primary care including BEmONC. Total health spending in the DRC was estimated at just $21 USD per person in 2019, substantially below the low-income country average of $34 and the sub-Saharan African average of $85, with out-of-pocket spending accounting for 40% of total expenditure. ^13^ The DRC faces a critical shortage of health workers, with an estimated 0.35 medical doctors per 10,000 inhabitants ^19^ which is substantially below WHO recommendations. Midwives are also in short supply, concerning given evidence suggests they could reduce maternal mortality by 67% and neonatal mortality by 64%. ^18^

Empirical data on the actual capacity and performance of health facilities delivering EmONC in the DRC remains lacking, and those studies that have been done paint a concerning picture. Casey et al. (2009, 2015) identified major gaps in reproductive health service delivery across DRC facilities, with most unable to meet basic EmONC standards. ^21, 22^ Ntambue et al. (2011) found similar deficiencies in the city of Lubumbashi, with critical shortages of trained staff, equipment and medicines. ^23^ More recently, Mizerero et al. (2021) conducted a facility-level assessment across three provinces in eastern DRC, again finding that most facilities failed to meet basic EmONC criteria. ^20^ These studies suggest that inadequate EmONC provision is a persistent and widespread problem in the DRC, yet little progress has been made in addressing it. Importantly, there remains a near-total absence of facility-level assessment data from rural and remote health zones, leaving policymakers without the empirical evidence needed to make informed decisions on resource allocation and investment. To address this issue, we conducted this study: to gather empirical data and assess the quality of emergency obstetric and neonatal care (EmONC) provided in all 63 healthcare facilities (excluding the general referral hospital) in Vanga district, Kwilu province, DRC.

## 2. Methods

### 2.1 Context

This study was conducted in the Vanga health zone, Kwilu province, where one lead author is a practicing doctor with understanding of the healthcare network, local geography, language and customs, enabling successful access to facilities and available data. Pregnant women represent 4% of the population. ^10^ The Vanga health zone is reported as containing 65 health facilities and one general referral hospital serving 43 health areas, the remotest of which is 90 km from the central town and first referral hospital in Vanga. However, two health facilities were found to be non-existent, leaving 63 health facilities and one hospital serving 119 villages. We excluded Vanga general referral hospital because it operates at a different care level, providing tertiary care, not primary EmONC. As a separate category, it was not in the focus or aims of this study.

The DRC has published guidelines for the provision of mother and newborn care. These define health facility requirements in the domains of human resources, infrastructure, materials, medicines and other tools, to provide quality care, see Table 1. These guidelines divide health facilities as “lower level” and “higher level” corresponding to the services they are expected to provide (basic or comprehensive EmONC), each with different requirements. However, facilities in Vanga are not formally categorised. 58 health centres do not employ a medical doctor and are expected to deliver basic EmONC (see Table 2), we group these as “Nurse-led” facilities. Five health facilities in Vanga employ at least one medical doctor and are expected to deliver the higher level of comprehensive EmONC (to include blood transfusions and caesarian sections) (see Table 2). We group these as “Doctor-led” facilities.

**Table 1.** DRC ministry of public health 2012 set of minimum infrastructure, human resource and material requirements for healthcare facilities. See full document in supplementary materials.

	Minimum Requirements for the Delivery of Qualified Assistance at Childbirth as per DRC ministry of public health guidelines
Intervention standard domain	Health centre
**Intervention standards**	12 standards to include use of a partograph, essential care for the newborn and timely referral
**Human resource standards**	Specific personnel standards in include two midwives on duty
**Infrastructure standards**	12 standards to include labour room and treatment room
**Material resource standards**	74 standards to include specific equipment such as a light source and examination table, medications such as antibiotics and antihypertensives and consumables such as sterile gloves and chlorine-based disinfectant

**Table 2.** WHO EmONC signal functions, adapted from WHO MoNITOR 2009. ^16^

Basic EmONC (bEmONC) signal functions, expected from all facilities	Comprehensive EmONC (cEmONC) signal functions, expected from only general referral hospitals (higher level care centres)
***Perform 1–7 signal functions***	***Perform 1–7 signal functions and 8–9***
(1) Administer parenteral antibiotics	(8) Perform surgery, e.g. caesarean section
(2) Administer uterotonic drugs	(9) Perform blood transfusion
(3) Administer parenteral anticonvulsants for pre-eclampsia and eclampsia	
(4) Manually removal of the placenta	
(5) Removal of retained products of conception	
(6) Perform assisted vaginal delivery	
(7) Perform basic neonatal resuscitation	

Vanga health zone has been part of the World Bank’s performance-based financing program since 2016. ^11^ Part of this program aims to improve healthcare access and quality for mothers and newborns. Under the program, facilities receive payments every three months based on the number and quality of services they provide, focusing on maternal, newborn, child, and reproductive health care. Quality is assessed using a standardised checklist. Two types of contract are used: 1) the minimum package of activities (MPA) for core preventive and curative primary health services and, 2) the complementary package of activities (CPA) for services delivered at first-level referral centres, e.g. complicated deliveries, blood transfusions and surgeries requiring anaesthesia. There are 18 MPA service indicators across maternal and childhood care. Examples include (with reimbursement values converted to $USD): deliveries attended by skilled birth attendants with filled partographs ($5.52) or postnatal consultation between three and seven days post delivery ($1.20). ^11^ There are 22 CPA service indicators, for example: uncomplicated delivery ($9) and caesarian sections ($42). Payments are subject to quality weighting adjustments calculated by standardized tools. Maternity quality indicators are weighted at 12% and include correct use of partogram, availability of supplies, cleanliness and confidentiality. ^11^

### 2.2 Study design

***Overview***

We conducted a cross-sectional survey of all Vanga health zone facilities excluding the general referral hospital between September and December 2023. Data in three domains was collected at each facility:

1. Caseload registry data from two periods: Jan-March 2021 and Jan-March 2022.
2. Facility infrastructure, human resource and material data was collected using an inventory checklist.
3. Signal function data and type of case handled (in the last twelve months) was collected through a structured questionnaire and registries.

**Figure 1.**
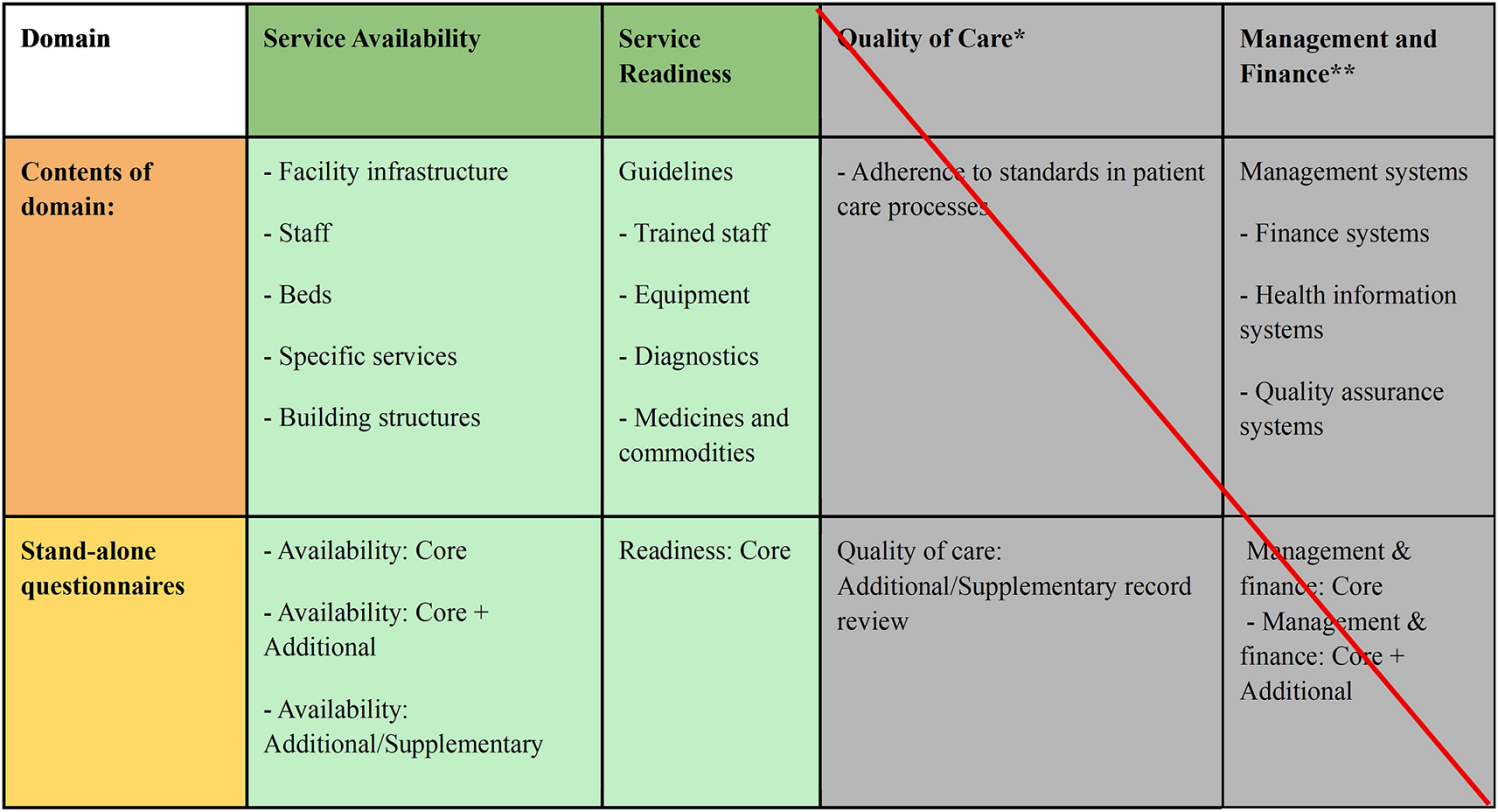
Overview of WHO HHFA modules and questionnaires used by the authors to provide information on the availability and quality of obstetric and neonatal healthcare within the Vanga health zone. Adapted from WHO HHFA Quick Guide 2022. ^11^ *Caseload data taken from facility registers was the intended method of assessing quality-of-care domain (indicators taken as proxies for adherence to care standards including use of partographs and complication rates), however, due to widespread suspicions of falsification of records, the data was unreliable and therefore was excluded from analysis.**Management and finance were deemed beyond the scope of the paper and is not included.

Several validated tools exist for standardised facility assessment, including the EmONC framework, ^6^ the Service Provision Assessment (SPA), developed and conducted by the DHS program ^7^ and the World Health Organisation’s Service Availability and Readiness Assessment (WHO SARA). ^8^ Data was collected using a French-speaking questionnaire developed and used with permission from Habonimana et al. 2022 ^15^ that used the WHO 2022 Harmonised Health Facility Assessment tool (HHFA) ^14^ as the primary framework. We used DRC Ministry of Public Health guidelines to contextualise the checklist and focus on the relevant items. Our survey was more focused in scope than the full HHFA tool and did not extend to examining management and financing. See Figure 1 for a schematic overview of domains assessed.

1. Caseload data

We collected caseload data from each facility for two 3-month periods from Jan-March 2021 and Jan-March 2022. Categories of data collected included number of live births, vaginal deliveries, emergency caesarean sections, transfusions, referrals to doctor-led and nurse-led facilities and maternal and neonatal complications. This data was intended to act as a proxy measure of quality of care, with metrics such as use of partographs and complication rates providing insight to adherence to care standards. Data quality was assessed by comparing records across two collection periods within each facility, and if values for multiple indicators were identical or near identical in both periods, this was considered indicative of possible falsification. Informal discussion with local staff was also considered when contextualizing these patterns. As we describe in 4.1, suspicions of widespread falsification of records meant data was deemed unreliable and could not be included for analysis.

2. Facility infrastructure, human resource and materials

To qualitatively assess resource availability aggregated across facilities (stratified as Doctor-led, CEmONC, or Nurse-led, BEmONC), we used a grading system adapted from Kosgei 2016. ^17^ For the 5 doctor-led facilities, if 5/5 had the indicator item present, it was graded “Excellent”, 4/5 “Good”, 3/5 “Moderate” and 2 or fewer/5 “Poor”. For the 58 Nurse-led facilities, If >90% facilities had the indicator item present, it was graded ‘“Excellent”, 89–75% “Good”, 74–50% “Moderate” and < 50% “Poor”. The decision was taken not to present aggregated indicators by individual facility to reflect the primary aim of the study, which was to characterise overall state of EmONC provision across Vanga, and assessing variation between individual facilities was deemed beyond the scope of the study. In addition, considering only 5 doctor-led facilities were included, there were concerns presenting aggregated data at the individual facility level risked indirect identification of centres.

3. EmONC signal functions

The EmONC signal functions are a set of nine clinical tracer interventions—seven basic (BEmONC) and two comprehensive (CEmONC) representing practices which evidence shows have the greatest impact in mitigating maternal and newborn morbidity and mortality. We utilised adapted survey tools built on the Donabedian model of assessing the quality of healthcare: evaluating structure, process and outcome. ^9^ We recorded EmONC signal functions at each facility. The 9 signal functions are summarised in Table Two. There are seven basic EmONC (BEmONC) signal functions and two comprehensive EmONC (CEmONC) signal functions. Signal functions are identified as the key medical interventions used to treat direct obstetric complications causing the vast majority of deaths worldwide. We assumed nurse-led facilities should perform BEmONC signal functions only while doctor-led facilities should perform all signal functions. Where facilities failed to perform an expected signal function we assessed why.

#### Data collection

Data collection was conducted by three collaborators familiar with local language, customs and culture working within the Vanga Health zone between September and December 2023. These were: the principal investigator (PI) (consultant physician); a trainee physician and a senior nurse. At each facility, one investigator spent an entire day collecting data. Rigorous steps were taken to ensure consistency in data collection: the PI trained the investigators on how to collect data using a standardized checklist and methodology. Equipment, supplies and medicines were identified and recorded at each facility and, where appropriate, checked to be in working order. Signal functions were assessed by collecting logbooks from the three months prior. To ensure consistency and minimise bias, the PI observed practice rounds whereby investigators were supervised collecting data. The investigators then independently collected data from a sample of facilities in duplicate with the PI so data could be evaluated for inconsistency. Throughout data collection, the PI sampled healthcare facilities to check the accuracy of data collection.

#### Data analysis

The data were presented in tabular form. Quantitative variables were described using the minimum, 25th percentile, median, 75th percentile, and maximum. Categorical variables were described using absolute frequencies and percentages. Statistical analysis of the data was performed using Stata/BE 18.5 and STATA/BE 19.5 software.

***Research ethics approval***

Ethical approval was obtained from the Comité National D’éthique De La Santé (the National Health Ethics Committee) of the Democratic Republic of Congo n°476/CNES/BN/PMMF/2023 on 25/08/2023. All procedures involving human participants were conducted in accordance with the ethical standards of the institutional and/or national research committee and with the 1964 Declaration of Helsinki and its later amendments. Written informed consent was obtained from all participants prior to participation (Healthcare facility staff who contributed to data collection through provision of facility records and structured interviews.) The consent form can be found in supplementary materials.

## 3. Results

### 3.1 Summary

Below we report key assessment findings for Doctor-led and Nurse-led facilities. Full data are provided in supplementary files A and B. Overall, broad system-level deficiencies were evident, with shortages of trained personnel, essential drugs, equipment, and a lack of both training and adherence to national guidelines and operating standards. EmONC signal function data - the WHO’s minimum standards of obstetric and neonatal care - revealed large deficiencies in the provision of core services. Signal function data also showed several facilities performed CEmONC services such as caesarian sections when not equipped with the trained staff required to provide these services safely, suggesting either that facilities were acting beyond their capability, or that service records are being falsified to access financial incentives (see Section 4.1 below).

Two of the 65 officially listed health centres were nonexistent when attempts were made to visit them and Vanga general referral hospital was not surveyed. We therefore surveyed 63 health centres over eight weeks between September and December 2023; 61/63 (97%) of facilities were public, 2/63 (3%) were private. 5/63 (8%) of facilities employed doctors (Doctor-led facilities), 58/63 (92%) did not (Nurse-led facilities).

### 3.2 Nurse-led facility caseload data from registers

The caseload data from the first quarter of the years 2021 and 2022 was often identical or nearly identical for all service categories within a specific facility, including number of live births and complications. This pattern was observed systematically across all 58 facilities, for example, the median number of live births was 52 in both periods (P25 18–20, P75 66–67) and median normal deliveries were 51–52 in both periods (P25 18–20, P75 67). Additionally, median figures for caesarean sections, referrals and complications were identical across both periods in the majority of facilities. Informal discussion with local service providers was used to contextualise these patterns.

### 3.3 Nurse-led facility infrastructure, human resource and material

Every health centre 58/58 (100%) reported being open 24 hrs a day seven days a week.

***Nurse-led facility basic infrastructure***

Only 2/58 centres (4%) had a reliable water source inside the facility and 16/58 (28%) had working latrines. Zero facilities had internet access though 54/58 facilities (93%) had access to a mobile phone owned by a health worker. 26/58 facilities (45%) used a solar system, the remaining 32/58 (55%) of facilities reported no means of electricity.

No facilities had access to ambulances for emergency transportation or stocked fuel supplies that could support patient transport. No facilities reported availability of blood storage refrigerators but 22/58 (38%) of facilities reported capability of blood screening for blood-borne disease prior to transfusion, suggesting any transfusion performed (a service not included in the BEmONC package) was linked to contemporaneous donation.

***Nurse-led facility bed capacity***

Nurse-led facilities had a median of 3 maternity beds (IQR, 1–4, max 8) and 0 delivery tables (IQR 0–0, max 2). No nurse-led facilities had specific neonatal areas, cots or incubators and none had a functional space for neonatal resuscitation. No facilities had national guidelines or training manuals for essential childbirth care available, and only 1/58 facilities (2%) had national guidelines or protocols for EmONC available in the facility.

***Nurse-led facility human resource***

The bulk of service delivery in nurse-led facilities is performed by nurses. The median number of nurses deployed to a facility and providing all services (including all outpatient care, for 24/7 service cover) was 4 (1–13). The median number of professional midwives at nurse-led facilities was 0 (IQR 0–1, max 2) while the median number of ‘matrons’ (equivalent to a healthcare assistant for maternity care who has not received any formal training) was 1 (IQR 0–1, max 2). All facilities reported routinely deploying at least one nurse/matron within the facility to offer maternity services including antenatal and delivery care. The median number of vaginal deliveries performed in the last three months was 31 (min 3, IQR 57–15, max 95).

Almost no facilities had access to trained laboratory technicians (median 0, IQR 0–0 max 1). Not all facilities employed cleaning staff (median 1 IQR 1–2, min 0, max 3).

We also assessed the qualification level and demographics of nursing staff. 81% of nurses were female (19% were male). The median age of nurses varied. See supplementary file B. All nurses and midwives reported at least one year of clinical experience (IQR 1–21, median: 9) and all had performed at least one delivery in the past three months (IQR 15–60, median: 32). The median years of experience service providers reported since completing their most recent professional qualification was 9 years (min 1, IQR 20–5, max 29).

***Staff training at Nurse-led facilities***

At any given facility, the maximum number of maternity service providers who had received training was 1. Only 11/58 facilities (19%) reported receiving EmONC training. All 11 facilities that had reported EmONC training had received training within the past two years.

In 34/58 nurse-led facilities (59%), service providers did however, report receiving some continuing education in maternal and neonatal health (consisting of family planning and antenatal care) since starting their employment. Only 8/58 (14%) nurse-led facilities reported receiving continuing education in BEmONC. Zero facilities reported receiving continuing education in CEmONC.

***Nurse-led availability of equipment, supplies and medicine***

Maternal and obstetric equipment

The data revealed stark deficiencies in the availability of equipment, supplies and medications across all facilities. Using the availability grading system adapted from Kosgei 2016 ^17^ (available in >90% facilities, Excellent; in 89–75%: Good; in 74–50%: Moderate and in less than 50%: Poor) the data shows poor overall availability of obstetric and maternal equipment. Only 3 of 21 items (two pairs of sterile gloves, two gynaecological disinfectants and a scissor/blade for cutting umbilical cord) were assessed as being “good” on the availability grading system. One item, blank partogram, was assessed “Moderate”, present in 31/58 (54%) facilities. 18 of the 21 items (86%) were assessed “Poor”. Across items assessed “Poor”, the median number of facilities stocking a maternal or obstetric item was 2/58 (4%) ( Table 3).

**Table 3.** Equipment, supplies and medications found in less than 50% of Nurse-led facilities.

**Indicator items classified poor (Found in < 50% Nurse-led facilities) by domain**
**Obstetric and maternal equipment (n = 23)**
Cord clamp 10/58 (17%) episiotomy scissors 12/58 (21%) Suture material with needle 24/58 (41%) needle holder 13/58 (22%) 2 sterile drapes 70 x 70 2/58 (4%) 2 x 5 sterile compresses 7.5 x 7.5 13/58 (21%) 2 3-ply masks 4/58 (7%) 2 jackets 4/58 (7%) 1 sterile newborn woad cannula t 000 1/58 (2%) 1 sterile pediatric suction probe ch 6 2/58 (4%) 1 biconical connector 2/58 (4%) 2 sterile umbilical clamps 1/58 (2%) 1 cap 2/58 (4%) 1 sterile isothermal sheet 1/58 (2%) 1 high concentration adult O2 inhalation mask 1/58 (2%) 1 very high concentration pediatric O2 inhalation mask 1/58 (2%) 1 pair of sterile scissors 11/58 (19%) Examination lamp 2/58 (4%) Anesthesia machine for delivering aesthetic gases and oxygen 0/58 (0%)
**Specialist neonatal care equipment (n = 16)**
Intubation Kit - Pediatric (complete with oropharyngeal airway, endotracheal tubes, laryngoscope, Magill forceps, stylet) 0/58 (0%) Suction cup/forceps 0/58 (0%) Fetal monitoring device 0/58 (0%) Ultrasound scanner 0/58 (0%) Pulse oximeter – pediatric 0/58 (0%) Pulse oximeter – neonatal 0/58 (0%) Phototherapy unit 0/58 (0%) Operating table 1/58 (2%) Baby scale in the delivery room 23/58 (40%) Blood pressure measuring device in the delivery room 10/58 (17%) Suction device (suction bulb or electric suction pump) 2/58 (4%) Neonatal bag and mask size 1 - for full-term babies 0/58 (0%) Neonatal bag and mask size 0 - for premature babies 0/58 (0%) Resuscitation table with heat source for newborn resuscitation 1/58 (2%)
**Medications (n = 11)**
Calcium gluconate 0/58 (0%) Hydralazine 0/58 (0%) Antibiotic eye ointment for newborns 0/58 (0%) Azithromycin (cap/tablet or oral liquid) 0/58 (0%) Magnesium sulfate 0/58 (0%) Misoprotol 1/58 (2%)

***Neonatal equipment***

Availability of specialist neonatal equipment was also poor. Only 1 of 15 items (sterile gloves) was assessed “Good”, available in 47/58, 81% of Nurse-led facilities; mirroring good availability of gloves for obstetric care. All other items, 14 of 15 (93%) were assessed “Poor”. The median number of facilities stocking a given neonatal item was 1/58 (2%). 8/15 neonatal care items (47%), were not stocked by a single facility ( Table 3).

***Medications***

Availability of medications was improved compared to other categories. Availability of 2/11 items were assessed as “Excellent”, gentamicin 56/58 (97%) and metronidazole 54/58 (93%). Ampicillin was assessed “Good” 45/58 (78%) while oxytocin 42/58 (72%) and sodium chloride (saline) solution 43/58 (74%) were both assessed “moderate”. Six of 11 items (55%) were assessed as “poor”. The median number of facilities stocking a given medication was 14/58 (24%). No nurse-led facilities stocked calcium gluconate or hydralazine ( Table 3).

### 3.4 Nurse-led facility EmONC signal functions

We collected data on the WHO’s EmONC signal functions across health facilities. Where facilities reported not performing a signal function we assessed reasons why. Findings are summarised in Table 4. The most commonly performed signal function was administration of parenteral uterotonics (54/58, 93%). Removal of retained products of conception was also performed by a large proportion of facilities (47/58, 81%). Just under half (28/58, 48%) reported administering parenteral antibiotics while a third (19/58, 33%) performed manual placental extraction. No facilities performed assisted delivery or neonatal resuscitation and only 1/58 facilities administered anticonvulsants for pre-eclampsia or eclampsia. The most widely cited reason for signal functions not being performed were supply chain and equipment issues and inadequate training.

**Table 4.** Doctor-led and B facility EmONC signal functions.

Signal function	Have parenteral antibiotics been administered to a pregnant or recently delivered woman within the past 3 months? (%)	Have any parenteral uterotonics been administered within the last 3 months? (e.g., parenteral oxytocin, ergometrine, misoprostol) (%)	Have anticonvulsants for severe preeclampsia or eclampsia been administered parenterally within the past 3 months? (e.g., magnesium sulfate) (%)	Has manual placental extraction been performed within the last 3 months? (%)	Has removal of retained products of conception been performed within the last 3 months? (e.g., manual vacuum aspiration, dilation and curettage) (%)	Has an assisted vaginal delivery (e.g., vacuum extraction, forceps delivery) been performed within the last 3 months? (%)	Has neonatal resuscitation with a bag and mask been performed in the last 3 months? (%)	Has a blood transfusion been performed in the last 3 months? (%)	Has a cesarean section been performed in the last 3 months? (%)
**Doctor-led facilities**									
**Done within the last 3 months? (Yes)**	5/5 (100)	5/5 (100)	0/5 (0)	2/5 (40)	5/5 (100)	0/5 (0)	1/5 (20)	5/5 (100)	5/5 (100)
**Not done in the last three months (No)**	0/5 (0)	0/5 (0)	5/5 (100)	3/5 (60)	0/0 (0)	5/5 (100)	4/5 (80)	0/5 (0)	0/5 (0)
**If not done in the last three months, why?**									
**Training issues**	0 (0)	0 (0)	0 (0)	2 (40)	0 (0)	0 (0)	0 (0)	0 (0)	0 (0)
**Supplies, equipment, medication issues**	0 (0)	0 (0)	1 (20)	0 (0)	0 (0)	5 (100)	4 (80)	0 (0)	0 (0)
**Management problems**	0 (0)	0 (0)	0 (0)	0 (0)	0 (0)	0 (0)	0 (0)	0 (0)	0 (0)
**Policy issues**	0 (0)	0 (0)	0 (0)	0 (0)	0 (0)	0 (0)	0 (0)	0 (0)	0 (0)
**No indication**	0 (0)	0 (0)	4 (80)	4 (80)	0 (0)	0 (0)	0 (0)	0 (0)	0 (0)
**Nurse-led facilities**									
**Done within the last 3 months (Yes)**	28/58 (48)	54/58 (93)	1/58 (2)	19/58 (33)	47/58 (81)	0/58 (0)	0/58 (0)	19/58 (33)	7/58 (12)
**Not done in the last 3 months (No)**	30/58 (52)	4/58 (7)	57/58 (98)	39/58 (67)	11/58 (19)	58/58 (100)	58/58 (100)	39/58 (67)	51/58 (88)
**If not done in the last three months, why not?**									
**Training problems**	3/30 (10)	2/4 (50)	14/57 (25)	7/39 (18)	17/58 (36)	24/58 (41)	40/58 (69)	17/39 (44)	7/51 (14)
**Supplies, equipment, medication issues**	19/30 (63)	4/4 (100)	36/57 (63)	7/39 (18)	27/58 (57)	53/58 (91)	50/58 (86)	27/39 (69)	6/51 (12)
**Management problems**	1/30 (3)	1/4 (25)	1/57 (2)	0/39 (0)	0/58 (0)	0/58 (0)	1/58 (2)	1/39 (3)	3/51 (6)
**Policy issues**	3/30 (10)	1/4 (25)	17/57 (30)	2/39 (5)	12/58 (26)	5/58 (9)	4/58 (7)	27/39 (69)	1/51 (2)
**No indication**	10/30 (33)	2/4 (50)	37/57 (65)	34/39 (87)	27/58 (57)	6/58 (10)	9/58 (16)	4/39 (10)	48/51 (94)

Despite not having medical doctors, a third (19/58) of nurse-led facilities reported performing blood transfusions, conducted without laboratory professionals’ support for cross-matching of products or proper blood product refrigeration capacity. Only 2/19 Nurse-led facilities performing transfusions reported no interruptions in the availability of blood products in the last 3 months. 7/58 Nurse-led facilities (12%) reported performing caesarean sections though they were only staffed by nurses.

### 3.5 Doctor-led facility caseload data

*Doctor-led caseload data*

Due to the falsification problem outlined in Section 4.1, a decision was made not to include caseload data.

### 3.6 Doctor-led facility infrastructure, human resource and materials

Doctor-led facilities are defined as those employing at least one medical doctor. Vanga referral centre was not included in the study.

***Doctor-led facility basic infrastructure***

One of five (20%) doctor-led facilities had access to a reliable water source powered by solar energy. Two of five (40%) had internet access. Five of five (100%) facilities had access to a private mobile phone. No doctor-led facility had access to ambulances for emergency transportation or fuel supplies. All five (100%) had electricity supply via solar systems. All facilities (100%) reported being open 24 hrs a day 7 days a week. Four of five facilities (80%) reported the capacity to screen blood products.

***Doctor-led facility bed capacity and environment***

Median total bed capacity was 22 (16, 19, 22, 22, 45), median maternity bed capacity was 7 (4, 7, 7, 9). 2 of 5 facilities had specific delivery beds. Despite performing caesarian sections, no doctor-led facilities had operating rooms or surgical beds. Caesarean sections were performed on patient gurneys. No doctor-led facilities had beds for newborn care, nor did any facility (doctor-led or B) provide basic neonatal care such as kangaroo mother care for premature/low birthweight babies or skin-to-skin contact support. No doctor-led facilities had specific neonatal areas, beds or incubators and none had a functional space for neonatal resuscitation. No doctor-led facilities had national guidelines, manuals or protocols for EmONC available on site.

***Doctor-led facility human resource***

Four of five doctor-led facilities had 12 nurses and one had 15 nurses (median 12). These staff provide all services including outpatient care for 24/7 care. The median age of nurses varied across facilities (see supplementary materials). Four of five facilities (80%) employed a single midwife. Two facilities (40%) employed 2 matrons (healthcare assistants who have not received formal training), one facility (20%) employed one matron and two facilities (40%) had zero matrons. No gynaecologists, paediatricians or neonatologists work in the Vanga healthzone. GPs (defined as medical graduates with no further postgraduate training) were the only medical specialty available, and only at the five Doctor-led facilities. Two facilities had 2 GPs, while three facilities each had 3 GPs. These doctors served the entire facility and were not dedicated to maternity care.

***Doctor-led availability of equipment, supplies and medicine***

Availability of equipment, supplies and medicine across doctor-led facilities was poor. We classified availability of items in 5/5 facilities as “Excellent”; in 4/5 as “Good”; in 3/5 as “moderate” and 2 or fewer out of 5 as “Poor”.

***Obstetric and maternal equipment***

The availability of obstetric and maternal equipment was inadequate. Only 5 of 23 (22%) items (two pairs of sterile gloves, sterile scissors, sterile compresses, blank partogram and disinfectant) were assessed “Excellent”. Availability of all other items in this category was “Poor” (see Table 5). 2/23 (9%) maternal and obstetric items (sterile isothermal sheet, adult high concentration O2 mask) were stocked by 0 facilities.

**Table 5.** Equipment, supplies and medications found in less than 50% of Doctor-led facilities.

**Indicator items classified as poor (Found in < 50% Doctor-led facilities) by domain**
**Obstetric and maternal equipment (n = 23) (%)**
Examination lamp 1/5 (20) Suction device 1/5 (20) Epistiotomy scissors 1/5 (20) Needle holder 2/5 (40) 2x Sterile drapes 1/5 (20) 2x Sterile 3-ply masks 1/5 (20) 2x sterile jackets 1/5 (20) 1 cap 1/5 (20) 1 sterile isothermal sheet 0/5 (0) high concentration adult O2 inhalation mask 0/5 (0) Blank partogram 1/5 (20) Operating table 1/5 (20)
**Specialist neonatal care equipment (n = 18) (%)**
1 sterile newborn woad cannula t 000 1/5 (20) 1 sterile pediatric suction probe ch 6 1/5 (20) 1 biconical connector 1/5 (20) Anesthesia machine for delivering aesthetic gases and oxygen 0/5 (0) Intubation Kit - Pediatric (complete with oropharyngeal airway, endotracheal tubes, laryngoscope, Magill forceps, stylet) 0/5 (0) 1 very high concentration pediatric O2 inhalation mask 1/5 (20) 2 sterile umbilical clamps 0/5 (0) Suction cup/forceps 0/5 (0) Fetal monitoring device 0/5 (0) Ultrasound scanner 0/5 (0) Pulse oximeter – pediatric 0/5 (0) Pulse oximeter – neonatal 0/5 (0) Phototherapy unit 0/5 (0) Baby scale in the delivery room 2/5 (40) Blood pressure measuring device in the delivery room 2/5 (40) Suction device (suction bulb or electric suction pump) 1/5 (20) Neonatal bag and mask size 1 - for full-term babies 0/5 (0) Neonatal bag and mask size 0 - for premature babies 0/5 (0) Resuscitation table with heat source for newborn resuscitation 0/5 (0)
**Medications (n = 11)**
Calcium gluconate 0/5 (0) Hydralazine 0/5 (0) Antibiotic eye ointment for newborns 0/5 (0) Azithromycin (cap/tablet or oral liquid) 0/5 (0) Magnesium sulfate 0/5 (0) Misoprotol 2/5 (40) Sodium chloride solution (injection) 1/5 (20)

***Specialist neonatal equipment***

Availability of specialist neonatal equipment was very poor. Only 2 of 18 items (11%) (sterile gloves, and baby weighing scales) were assessed “Excellent”. All other neonatal items, 14 of 18 (78%) were assessed “Poor”. 12/18 neonatal care items (67%) were not available in any facility.

***Medications***

Availability of medications was improved compared with other categories. Three items were assessed “Excellent” (gentamicin, metronidazole and ampicillin) stocked by all five facilities. Oxytocin (4/5 80%) was assessed “Good”. Six of 11 items (55%) were assessed “Poor”. No nurse-led facilities stocked calcium gluconate, hydralazine or magnesium sulfate (3/11 27%).

***Doctor-led facility staff training and experience***

Four of five type (80%) Doctor-led facilities had received BEmONC training. Zero facilities had received CEmONC training. Four of 5 (80%) facilities also received continuing education in maternal and neonatal health to include family planning and antenatal care.

### 3.7 Doctor-led facility EmONC signal functions

EmONC signal function data collected from Doctor-led facilities is summarised in Table 4. All Doctor-led facilities reported performing the BEmONC signal functions of administering parenteral antibiotics, uterotonics and removal of retained products of conception. However, none reported administering anticonvulsants or performing assisted vaginal delivery and only two and one Doctor-led facility reported performing manual removal of the placenta and neonatal resuscitation respectively. Conversely all five had performed both blood transfusion and caesarian section, the CEmONC signal functions, in the previous 3 months.

## 4. Discussion

### 4.1 Summary

We surveyed the 63 local health facilities operating within Vanga healthzone (excluding Vanga general referral hospital), Kwilu province, DRC to assess the quality of care provided to mothers and newborns. While ‘on paper’, the population served by the Vanga health zone may appear to have good access to maternal and neonatal care (63 health facilities and 1 general referral hospital for a population of approximately ~362,465), ^10^ our new data suggests this to be misleading due to severe inadequacies across all domains assessed. These results reveal that the current levels of infrastructure, human resource, equipment, supplies, medicines do not display a readiness to provide BEmONC, or in Doctor-led facilities, CEmONC as outlined by the WHO or local guidelines.

Furthermore, our data raises concerns over widespread implementation of a number of dangerous practices that may be causing harm to patients, including unsafe blood transfusions (in both doctor-led and B facilities) and caesarean sections performed in the absence of doctors, equipment or operating theatres.

Our study has also raised questions over the effectiveness and possible harms of pay-for-service schemes through incentivisation of falsification of results and we suggest future schemes should include more quality-weighting.

**Caseload data**

The caseload data analysed was so similar across the two collection periods that it was incredibly unlikely to be due to chance, with all summary statistics reading identical or near-identical. It was postulated, alongside informal discussions with local care providers, that records were being falsified to maintain adherence to P4P incentivisation schemes in place. These findings should be viewed in context: many, if not all centres, are reliant on such schemes for financial survival and as these centres are the only facilities providing EmONC services for the communities they serve. The falsification does not necessarily reflect ill intent.

### 4.2 Infrastructure, availability of equipment, supplies and medicine

The lack of availability of basic infrastructure and resources suggests obstetric and neonatal care within the Vanga healthzone is dangerously undersupported and underequipped, raising serious concerns of unsafe practice and patient harm. Almost all (61/63, 95%) of health facilities have no source of water, more than half have no electricity and those that do rely on solar power. There was no capacity to transfer patients, rendering access to higher care non-existent. Only 2/63 facilities had internet access only and 59/63 (94%) facilities had access to a mobile phone, meaning access to emergency consultation or guidelines is virtually non-existent. There is also evidence of unsafe practice. Zero nurse-led facilities reported blood refrigeration capacity, yet 19/58 report performing transfusions, suggesting any transfusion performed (a service not included in the BEmONC package) was linked to contemporaneous donation. This practice outside of the cold-chain risks the viability of blood products and greater infection risk, especially due to the absence of trained laboratory staff, suggesting that adequate cross-matching is not taking place.

Bed capacity was inadequate, with near-zero delivery tables (median 0, IQR 0–0). This suggests that deliveries and in some cases, caesarian sections, were being carried out in beds not clinically fit for purpose.

The deficiencies outlined here go beyond simple underfunding but suggest structural failing in the governance of the Vanga health system. Shortages of equipment or essential medicines in every single centre assessed suggests possible problems with procurement or distribution of resources, and the fact that two officially listed facilities were found not to exist on examination is evidence of a disconnect between information available at government level and the situation on the ground. Structural and governance problems will need to be resolved to make a meaning impact on improving quality of EmONC provision in the health zone.

### 4.3 Human resource concerns

Our results revealed major human resource constraints on service delivery in the Vanga health zone. Service delivery is reliant upon nurses, and there was at least one nurse at each health facility (median 6 (min 1- max 15) nurses at doctor-led facilities, median 2 (min 1- max 4) at nurse-led facilities). There were few midwives and the presence of doctors was rare, limited to 13 physicians operating across 5 health centres. The data suggests staff are working vastly extended hours, or facilities are in fact not offering a full range of services/hours. In centres employing 1–2 nurses, where sickness/absence occurs, service delivery is presumably constrained. There were zero specialists working in the health zone, with all physicians being ‘GPs’ - defined as medical graduates with no postgraduate training. These figures represent the total staff numbers for health centres and, as all 63 facilities purport to offer 24/7 care.

There were several major patient safety concerns raised by the data collected. Human resource data revealed widespread evidence of staff acting beyond their official competence or capability. For example, 7/58 nurse-led facilities report performing caesarian sections and 18/58 nurse-led facilities report performing blood transfusions, both without any doctors present. These nurses have not received formal training in performing caesarian sections, instead obtaining experience from shadowing colleagues. These nurses are also responsible for use of the anaesthetic (ketamine), again without formal training. However, when assessing evidence of unsafe interventions performed, it is important to recognise the lack of alternative options: zero facilities had access to ambulances to transfer patients, and many staff may have been driven by desire to prevent patient deaths in the absence of any alternatives.

Our findings reflect the broader DRC workforce crisis, with an estimated 0.35 medical doctors per 10,000 inhabitants nationally. ^19^ Geographical isolation and a lack of basic infrastructure likely exacerbates recruitment difficulties in rural zones like Vanga. The DRC’s ability to improve its recruitment and allocation of skilled healthcare staff while it addresses basic infrastructure and resource shortages will be crucial to any effort to improve its obstetric and neonatal patient outcomes.

### 4.4 EMONC signal functions and safety concerns

There is a lack of training for healthcare staff in the provision of EmONC and a large number of unsafe practices. For example, less than 30% of healthcare staff involved in childbirth have received formal training in emergency obstetric and neonatal care. Perhaps as a result and reflecting absence of equipment, only 5% of facilities reported offering assisted vaginal delivery and very few had experience or capacity to administer anticonvulsants to women with pre-eclampsia/eclampsia. In contrast, blood transfusion was reported in more facilities than one might expect, with the majority of those performing blood transfusion not able to screen for infectious diseases or even perform cross-matching prior to transfusion.

There are particular concerns over readiness to offer essential forms of neonatal care. No facilities had a dedicated space and equipment for resuscitating newborn babies. Only one of 63 facilities (a Doctor-led facility) reported performing neonatal resuscitation, and equipment or medicines for providing this or other forms of newborn care were almost universally non-existent in health facilities.

### 4.5 Comparison with existing literature

Our study was limited to the single health zone of Vanga in Kwilu province, south-western DRC, however the deficiencies identified are unlikely to be isolated and have been documented across many other provinces in the country. Other facility assessments conducted in the DRC have drawn similar conclusions, ^20– 23^ and the geography, population and infrastructural challenges identified in Vanga are generalisable a far larger part of rural DRC. However, the near-total absence of equivalent studies in the literature that report granular data on an indicator-by-indicator basis makes direct comparison difficult, mandating further investigations of this kind.

Our results also question the effectiveness of pay for performance (P4P) schemes in the DRC. While evidence suggests such schemes may improve antenatal care in the DRC, ^24^ evidence on benefits to neonatal and maternal health outcomes is less convincing. ^24^ Our study suggests P4P is conceivably being abused through falsification of patient records to achieve financial rewards. This is especially damaging given the already critical shortage of reliable health data from health facilities within the DRC. These findings echo a growing body of evidence raising questions over such incentive schemes. ^15^ More evidence now supports the transition from purely P4P schemes to “quality-adjusted models” that combine the incentivization of both volume of procedures and quality metrics. Huillery and Seban et al. 2021 identified incentivising volume alone can lead to “gaming”, whereas adding quality audits and tying payment to protocol adherence resulted in improved clinical outcomes. ^29^ Similarly, Basinga et al 2011 demonstrated that when P4P models implemented in Rwanda incorporated quality within the payment structure, quality of prenatal care improved. ^27^ A 2021 Cochrane review including 59 studies evaluating P4P in LMICs found schemes using payment per output adjusted for service quality had the largest positive effect on patient outcome. ^28^ Our findings, taken with the existing literature, highlight problems with traditional P4P schemes and suggest focus should be shifted towards quality-adjusted value-based-care.

### 4.6 Limitations

Due to concerns over the credibility of the caseload data, we cannot relate facility survey data on resources and care provision to facility caseload data. Additionally, there was a substantial time difference between survey data (2023) and registry data (2021–2022), introducing temporal inconsistencies when evaluating findings. Consequently, we cannot link shortages in specific areas to patient outcomes. The cross-sectional nature of the study means we are unable to follow trends in facility services and resources over time. The study included all health facilities (excluding Vanga referral hospital) within the Vanga health zone, a single district in Kilwu province. This limits geographical generalisability of conclusions to the wider DRC.

We identify potential sources of observer and response bias: data collection was conducted by healthcare workers operating within the health zone, possibly known to participants, which may have influenced responses, especially for sensitive indicators. We made attempts to minimise this risk by cross-checking reported data with logbooks and by utilising a rigorous data collection protocol, outlined in Methods.

The grading system used to assess indicator availability was adapted from Kosgei et al. ^17^ and has not been formally validated. The thresholds applied are pragmatic cutoffs and findings should be interpreted with this in mind.

Our assessment of data quality relied on observed patterns of implausible consistency between time periods instead of formal statistical testing. Methods that would have strengthened this argument include the Runs test for non-randomness or systematic variance analysis, which would provide a far stronger basis for conclusions about data-falsification.

Despite these limitations, we provide evidence of major patient safety concerns and comprehensive data on current infrastructure and operational status of healthcare facilities within the Vanga health zone, DRC. This previously unknown information should be used to inform the challenging process of decision-making on allocation of material and human resources, the need for health worker training, and investments in basic infrastructure in the region.

### 4.7 Future directions

There is a substantial lack of comparable studies in the current body of literature providing granular detail data from healthcare facilities operating in remote and rural DRC, which would be imperative to identify if the problems and deficiencies identified in this study are truly reflective of a wider, country-wide story. Future reports of this kind should publish time and financial costs incurred during the process of investigation and analysis, to provide funding bodies and researchers more information to allocate resources and ensure the feasibility of similar work in the future.

Improving the validity of collected facility data could be achieved by introducing routine data quality checks using statistical tests to evaluate the credibility of caseload data.

Alternative approaches to facility financing and their effects on maternal and neonatal outcomes should also be examined given the concerns over P4P schemes in use in rural DRC. These should be P4P schemes that incorporate quality measures, not just rewarding volume of service provided (e.g. use of partograph, appropriate referral). As more attention turns to the development of broader integrated primary care systems that include EmONC, targeted healthcare worker training programs focusing on specific signal functions that have been identified as priorities (e.g. anticonvulsant administration and basic neonatal resuscitation) should be introduced, and changes to training should be made to make it less attendance/experience driven and more competence-assessed.

## 5. Conclusion

There is evidence of major deficiency in the quality of EmONC services provided to mothers and babies in the Vanga health zone, Kwilu province, DRC, in every domain assessed. Facilities lack basic infrastructure and are not resourced with the materials or staff to provide adequate EmONC. Many facilities do not perform basic EmONC services, while others perform comprehensive EmONC services such as caesarian sections, blood transfusions and anaesthesia without appropriate staff, training or equipment present. Caseload data is invalid due to suspicions of falsification of records for financial incentives. Taken together, we present evidence that women and babies in the Vanga healthzone are likely suffering avoidable morbidity and mortality through insufficient access to EmONC, and potential harm from dangerous practices.

### Ethics and consent

Ethics approval was received from the Comité National D’éthique De La Santé (the National Health Ethics Committee) of the Democratic Republic of Congo n°476/CNES/BN/PMMF/2023 on 25/08/2023. Written informed consent was obtained from all participants prior to participation.

### Data availability statement

The full dataset contains information of a potentially sensitive nature, including evidence of deviation from national guidelines and irregularities in recordkeeping. Due to the small number of facilities within a clearly defined region where data was collected from, there is a real risk of indirect identification of facilities or staff. Ethical approval for the study was granted on the condition that individual-level data would not be made publicly accessible, and for this reason, the full dataset cannot be made freely available through an open-access repository. However, data may be shared upon request by contacting lead author Dr. Junior Mudji, mudjijunior@gmail.com, in accordance with ethical and confidentiality requirements.

### Extended data

Figshare. Facility-Based Assessment of Emergency Obstetric and Neonatal Care in Vanga Health Zone, Kwilu Province, Democratic Republic of Congo https://doi.org/10.6084/m9.figshare.30712898 ^26^

This project contains the following underlying data:

• Figures and Tables. (A full set of included figures and tables).
• Study Proposal. (A copy of the original study proposal).
• Supplementary file A. (A full set of data collected from Doctor-led facilities).
• Supplementary file B. (A full set of data collected from Nurse-led facilities).

Data is available under the terms of the CC BY 4.0.
