# Supplementary material for: Facility-Based Assessment of Emergency Obstetric and Neonatal Care in Vanga Health Zone, Kwilu Province, Democratic Republic of Congo
Source: Wellcome Open Res. 2026 Apr 27;11:1. Originally published 2026 Jan 2. [Version 2] doi: 10.12688/wellcomeopenres.25137.2 (PMC13014088; doi:10.12688/wellcomeopenres.25137.2)
Supplement: Peer review history

# Reviewer report: Reviewer response for version 1

**DOI:** 10.21956/wellcomeopenres.27710.r149936

**Published:** 2026-03-24

**Authors:** Zoaib Habib Tharwani

**Affiliations:** 1 Mayo Clinic Florida, Jacksonville, Florida, USA

Abstract:

1. Abstract mentions data collection timeline as Jan-March 2021 and Jan-March 2022; however, Methodology section mentions that surveys were done in 2023.
2. Typographical error: First word in methods section of Abstract should be capitalized.

Introduction:

1. Rationale could be better justified, and research gap should be expanded to increase impact.
2. Justification for selecting Vanga health zone should be made stronger.

Methods:

1. There are some hints towards selection bias. Authors should address that or provide robust reasoning.
2. The grading system, i.e. excellent, good, etc., is not justified, validation is needed.
3. It is mentioned that written informed consent was obtained from participants, but is this relevant to the current study design?

Results:

1. In some areas results are written in a manner which points towards interpretation which should essentially be a part of discussion section as opposed to the results section.
2. Multiple paragraphs describe item-level inventory data which can be put into supplementary tables.
3. Current tables appear very congested. Edits should be made to make them presentable and easier to interpret.

Discussion:

1. The tone of discussion should be polite and consistent. In some places it appears to be accusatory.
2. Structural causes apart from resource limitation are not discussed such as governance issues, supply chain problems, etc.
3. Limitations could be more descriptive and could include observer and measurement bias, mismatch between survey data, 2023, and registry data, 2021-2022, and lack of data on patient outcome.
4. In future directions section the components seem general. Authors could try to make it more specific like including workforce training programs or audits for reporting etc.

Several grammatical, typographical, sentence spelling errors are present throughout the manuscript.

Is the work clearly and accurately presented and does it cite the current literature?

Yes

If applicable, is the statistical analysis and its interpretation appropriate?

I cannot comment. A qualified statistician is required.

Are all the source data underlying the results available to ensure full reproducibility?

Yes

Is the study design appropriate and is the work technically sound?

Yes

Are the conclusions drawn adequately supported by the results?

Yes

Are sufficient details of methods and analysis provided to allow replication by others?

Yes

Reviewer Expertise:

Maternal and Infant health; Infectious Diseases and Epidemiology; Neurology and Neurosciences

I confirm that I have read this submission and believe that I have an appropriate level of expertise to confirm that it is of an acceptable scientific standard, however I have significant reservations, as outlined above.

# Response

**Published:** 2026-04-01

**Authors:** Kieran Desmond

**Affiliations:** Not Applicable, UK

We thank you for your detailed review. The comments have helped us strengthen several aspects of the manuscript including the Methods, Results and Discussion sections. We address each comment in turn below.  

**COMMENT **1 Abstract mentions data collection timeline as Jan-March 2021 and Jan-March 2022; however, Methodology section mentions that surveys were done in 2023. Typographical error: First word in methods section of Abstract should be capitalized.  

**AUTHOR RESPONSE** Thank you for identifying these errors. The typographical error was resolved.  We agree that the Abstract was unclear in separating the two data collection activities: the cross-sectional survey was conducted in sept-dec 2023, with the retrospective collection of caseload data from jan-march 2021 and jan-march 2022. We amended the abstract to make this wording clearly reflect this.   

**COMMENT **2 Introduction: Rationale could be better justified, and the research gap should be expanded to increase impact. Justification for selecting Vanga health zone should be made stronger.  

**AUTHOR RESPONSE** We agree the original introduction could have been strengthened including the justification of the rationale and the research gap. The revised manuscript provides a more detailed account of the research gap and a more rigorous justification of the selection of Vanga and why this study was warranted.   

**COMMENT **3 Methods: There are some hints towards selection bias. Authors should address that or provide robust reasoning. The grading system, i.e. excellent, good, etc., is not justified, validation is needed. It is mentioned that written informed consent was obtained from participants, but is this relevant to the current study design?  

**AUTHOR RESPONSE** We agree that the methods section outlining collection of consent was misleading and poorly written. We amended the section to explicitly outline that the consent was collected not from patients (as no patients were enrolled in the study) but instead from the healthcare facility staff that contributed in the form of structured interviews and providing facility registers.    We agree that potential sources of bias presented by the nature of the methodology where not sufficiently addressed in the original manuscript. We have now outlined the specific risks to validity in the limitations section of the discussion to include concerns regarding the openness/accuracy of responses considering the investigators were known healthcare workers within the health zone. In the revised manuscript we provide more robust reasoning, pointing to the rigorous data collection protocol used and the cross-checking of data.    We acknowledge the comment regarding the use of a grading system that is not validated. We intended for it to be used solely as a descriptive aid, rather than a formally validated instrument, and we ensured the raw data was also made available in the paper alongside. We have added a comment in the limitations section to reflect this, and will bring this comment into consideration in future works when considering use of validated tools.   

**COMMENT 4** Results: In some areas results are written in a manner which points towards interpretation which should essentially be a part of the discussion section as opposed to the results section. Multiple paragraphs describe item-level inventory data which can be put into supplementary tables. Current tables appear very congested. Edits should be made to make them presentable and easier to interpret.

**AUTHOR RESPONSE** We agree with the reviewer’s comment that in several parts of the results comments were of an interpretive nature, and should have been relocated to the discussion. We made several amendments to the results where this occurred, specifically, interpretations of the consistency of the caseload data, interpretations of contemporaneous transfusion based on the EmONC signal function and refrigerator indicator findings, interpretations of nurses performing caesarian sections, and interpretations around overworking and understaffing practices. In all these instances, the interpretations were removed and placed in the discussion.  We appreciate and acknowledge the critique of the granular indicator-level presentation of results. We used this approach because the primary aim of the study was to provide comprehensive facility-level data to inform targeted resource allocation decisions by policymakers and local health managers in Vanga and similar settings. We felt that moving item-level inventory data to supplementary tables would reduce the accessibility and actionability of the findings for this audience, who needed to see the specific items missing from facilities. We have taken several steps to improve the synthesising of findings, largely through providing summary layers over the granular data, for example the summary statistics added to Results 4.1. We also tried to highlight the most pertinent findings clearly in the summary section of the Discussion. We recognise that the current table formatting could be improved and in future will consider more carefully which tables need to remain in the main body of the text vs which ones should be moved to supplementary materials.   

**COMMENT **5 Discussion: The tone of discussion should be polite and consistent. In some places it appears to be accusatory. Structural causes apart from resource limitation are not discussed such as governance issues, supply chain problems, etc. Limitations could be more descriptive and could include observer and measurement bias, mismatch between survey data, 2023, and registry data, 2021-2022, and lack of data on patient outcome. In future directions section the components seem general. Authors could try to make it more specific like including workforce training programs or audits for reporting etc.  

**AUTHOR RESPONSE** We recognise the tone of the discussion in the original draft was inappropriate and have made substantial revisions, specifically to the section that explores the findings of procedures such as caesarian sections being performed by staff acting beyond their clinical competence. We acknowledge the incredible difficulty of taking life-and–death decisions to act or not in contexts where there is no referral capabilities, and urge readers to contextualise these findings as present due to system-wide failings, not individual negligence. We make similar revisions when discussing financial fraud.    We have amended the discussion to include a greater exploration of findings that are suggestive of governance issues and supply chain/distribution issues. We make several new comments and cite evidence from the results to support them. In doing so, the new discussion section provides more depth and insight. We argue that these structural elements must be corrected if meaningful change is to be achieved to EmONC provision in the health zone.    We have substantially added to the limitations section, to include sources of observer and measurement bias, the temporal inconsistencies introduced by the time lag between collection of survey and registry data and the lack of data on patient outcomes.   We substantially revised the future directions to move away from general, vague language towards specific interventions and improvements across a range of domains that are relevant to policy-makers, as based on the findings of the paper.

# Reviewer report: Reviewer response for version 1

**DOI:** 10.21956/wellcomeopenres.27710.r149934

**Published:** 2026-03-20

**Authors:** Diana Montoya-Williams

**Affiliations:** 1 The Children's Hospital of Philadelphia, Philadelphia, Pennsylvania, USA

This is an incredibly important landscape assessment of the obstetric and neonatal healthcare delivery system in a rural district in the DRC. While I am not as familiar with previously published literature of this kind in this area of the world, I recognize the incredible public health and health policy import of the data meticulously collected and well described in this paper. It is the kind of data that can lead to advocacy for policy change and structural solutions to the devastating care gaps the study authors found in the Vanga district. I have a few questions meant to strengthen this important work:

1. The authors found that some Type B facilities were providing C-sections. They hypothesize that “suggesting either that facilities were acting beyond their capability, or that service records are being falsified to access financial incentives.” They know the landscape and culture well, but it did feel like the first part of the sentence glosses over what could be very well-intentioned healthcare practices by people with few options. I do appreciate that the concept of “no alternatives” is raised on page 13 of the discussion. Part of me wonders whether the wording in the discussion could be enhanced to allude to the possibility that some providers did not want to let some women die and acted out of desperation. I worry that well-intentioned and tested to the limit providers in these health centers may read parts of this paper and not feel understood or seen when the messaging is mostly around fraud and potential harm caused. The authors are not wrong about the harm, but it seems like the authors could take the opportunity to also humanize these healthcare providers as part of their hypotheses-driven discussions for why C-sections occur where they shouldn’t. 
2. Similarly, I do think identifying financial fraud is critical, but could it be contextualized? This fraud may be the only thing keeping some of these centers alive as they clearly are in desperate need of economic investment. Is this the space to discuss that P4P models could instead choose to incentive performing on quality vs. on procedures? The various ways to operationalize Value based care is not mentioned but feels relevant in the discussion. 
3. Perhaps I missed it but why was Vanga general hospital not surveyed? Seems an odd healthcare system to leave out. Would just ensure that decision is justified in the methods.

Is the work clearly and accurately presented and does it cite the current literature?

Yes

If applicable, is the statistical analysis and its interpretation appropriate?

Yes

Are all the source data underlying the results available to ensure full reproducibility?

Yes

Is the study design appropriate and is the work technically sound?

Yes

Are the conclusions drawn adequately supported by the results?

Yes

Are sufficient details of methods and analysis provided to allow replication by others?

Yes

Reviewer Expertise:

I am a neonatologist who studies health disparities in the US by race, ethnicity and nativity with a focus on immigration policies on their impact on healthcare utilization and health outcomes.

I confirm that I have read this submission and believe that I have an appropriate level of expertise to confirm that it is of an acceptable scientific standard.

# Response

**Published:** 2026-04-01

**Authors:** Kieran Desmond

**Affiliations:** Not Applicable, UK

Thank you for your thoughtful and considered feedback, particularly the observations regarding the human context of the findings. These comments made us reflect on the framing of several key findings and we believe the revised Discussion is more balanced and appropriate as a result. We address each comment in turn below.  

**COMMENT** 1 The authors found that some Type B facilities were providing C-sections. They hypothesize that “suggesting either that facilities were acting beyond their capability, or that service records are being falsified to access financial incentives.” They know the landscape and culture well, but it did feel like the first part of the sentence glosses over what could be very well-intentioned healthcare practices by people with few options. I do appreciate that the concept of “no alternatives” is raised on page 13 of the discussion. Part of me wonders whether the wording in the discussion could be enhanced to allude to the possibility that some providers did not want to let some women die and acted out of desperation. I worry that well-intentioned and tested to the limit providers in these health centers may read parts of this paper and not feel understood or seen when the messaging is mostly around fraud and potential harm caused. The authors are not wrong about the harm, but it seems like the authors could take the opportunity to also humanize these healthcare providers as part of their hypotheses-driven discussions for why C-sections occur where they shouldn’t.   

**AUTHOR RESPONSE **  We agree that the original paper did not appropriately contextualise findings of individuals acting beyond their clinical competence and financial fraud. We have revised the relevant section of the results to briefly mention the weight of the decisions that are being made by health care workers on the ground, and entirely revised the discussion section where these findings are explored. The revised discussion explicitly states that the root cause should be identified as system-level failings, not individual negligence, and we have made attempts to provide a more human, sympathetic approach.   

**COMMENT **2 Similarly, I do think identifying financial fraud is critical, but could it be contextualized? This fraud may be the only thing keeping some of these centers alive as they clearly are in desperate need of economic investment. Is this the space to discuss that P4P models could instead choose to incentive performing on quality vs. on procedures? The various ways to operationalize Value based care is not mentioned but feels relevant in the discussion.   

**AUTHOR RESPONSE **We thank the author for this observation. We have added an additional section to the discussion titled “caseload data” where we contextualise the suspicions of caseload falsification with a more human approach. We have substantially revised the existing literature section of the discussion to include the evidence basis of the use of P4P programs with quality-adjusted incentives in the DRC and other comparable health systems. We point to studies which found that programs with quality-adjusted P4P elements yielded better patient outcomes to make the point that operationalized value based care should focus more on quality elements.   

**COMMENT **3 Perhaps I missed it but why was Vanga general hospital not surveyed? Seems an odd healthcare system to leave out. Would just ensure that decision is justified in the methods.  

**AUTHOR RESPONSE** We thank the reviewer for this comment and agree that the rationale for exclusion was not explicitly stated in the methods. We have adjusted 3.1 to include an explanation that the focus of the paper was on facilities offering primary EmONC, and as a tertiary centre, the referral hospital provides a different level of care beyond the focus of this paper.

# Reviewer report: Reviewer response for version 1

**DOI:** 10.21956/wellcomeopenres.27710.r145085

**Published:** 2026-02-13

**Authors:** Hannah H Leslie

**Affiliations:** 1 University of California San Francisco, San Francisco, California, USA

I appreciate the opportunity to review this manuscript, which provides detailed results from a census of health facilities within a single zone in the DRC and demonstrates concerning gaps in infrastructure throughout this area. Please see below my suggestions for strengthening the methods and results presented. 

 Introduction may be stronger with presentation of what a well functioning maternal and newborn care system looks like - e.g. capacity to do BEmONC and refer to facilities that can do CEmONC; specific inputs and processes to obtain desired patient outcomes - as well as the context of maternal health in DRC, and the similarity of Vanga to the rest of the country (including some of the setting information presented in methods). Results of prior health system assessments in DRC or (if applicable) any similar settings could be briefly mentioned. The details on how quality is measured and the suite of tools can be presented in the Methods section at the same time as this tool is defined. In other words, focusing the introduction on the What and Why questions and the Methods more on How would strengthen the article. 

 Study design: the three types of data collection should not be described as domains - distinguish between the ‘what’ - the quality domains and indicators prioritized - and the ‘how’ - the type of data used to assess these domains. In clarifying this, please provide greater clarity on whether the Quality of Care domain crossed out in Figure 1 was excluded from the tool or was intended to be addressed using the caseload data; typically case load data is insufficient to provide insights on adherence to standards, beyond perhaps ‘partograph used.’ Additional detail on the types of records that were intended to be used and how this mapped to quality of care (if part of the original study design) would be useful. It would also be helpful to consolidate the description of the survey tool, which is described as based on the HHFA but also drawn from a separate French-language assessment. If this is described in one location and with clarification on if the French tool was an adaptation of the HHFA and how it differed from the original, that would assist the reader

 Several terms and classifications are used that have the effect of making the results less rather than more clear, including ‘type A’ and ’type B’ facilities as well as the grading system of indicators; in both cases, the underlying facts of the facilities and the indicator level are more useful than the labels. Why not use meaningful labels like BEmONC and CEmONC? Or doctor-led and nurse-led? 

 Whatever label, consider presenting the two tiers in the same order throughout - right now Type B comes first in the results but second elsewhere. 

 Level of analysis: the majority of the results are presented by indicator for the facility tier, as opposed to aggregating by facility to consider the readiness of each facility overall. This may be a reasonable choice given the goal of describing Vanga Zone as a whole (minus the referral hospital). This choice should be stated explicitly in the Methods; I would also consider summing up indicators within facilities as a way of providing insight on how much the facilities vary within the overall very low level of inputs to care identified. 

 Case load data and falsification: This is an important finding that is currently split between the methods and results, weakening its clarity and impact. The methods of case load data extraction and data quality assessment should be reported in the Methods section, and the findings and conclusion that the data are not reliable should be a key aspect of the Results section. For data quality checks, consider WHO data standards for HMIS as one resource https://www.who.int/data/data-collection-tools/health-service-data/data-quality-assurance-dqa, although these focus on too much variability rather than too little; other tests such as the Runs test to check for non-randomness may be helpful, as would reporting variance and plotting the data to show the patterns. It’s actually an interesting oversight in the data quality assurance process, and one that could make its own analysis, considering for instance expected variation in facility data as drawn from sources like SPA to inform standards. That’s beyond the scope of this paper, but I think a basic assessment of the data is well within the scope of this analysis and would be a valuable contribution. 

 The findings at present are quite granular, and could benefit from additional focus and synthesis to bring out key points. In addition to aggregating by facility, it may be helpful to consider use of the figures to illustrate the findings and highlight gaps. These could be basic column or bar plots to show frequency of the items found (ordered by frequency to highlight the least frequent) or heat maps / color coded tables to highlight gaps. See for example Yang et al, Antenatal corticosteroids in low- and middle-income countries (JOGH, 2025 doi: 10.7189/jogh.15.04149 ) for examples of data presentation that could be considered. 

 The results and discussion clearly draw attention to the major challenges in infrastructure within the Zone; can additional information be provided to contextualize this within DRC and suggest whether similar conditions are likely in other areas of the country? I understand this is a detailed assessment of all facilities within one zone and is not intended to represent the country as a whole, but some context to understand whether this zone is likely to be an extreme outlier or fairly similar to other rural areas would be helpful. Potentially outside the scope of this article, but understanding the time and financial cost of this assessment would be interesting in terms of considering what types of routine and in-depth assessment the country or regions within the country might undertake, given the extreme deficits identified and the need to prioritize resources for system strengthening. 

 Minor notes

 - Consider including a map to orient readers to Vanga Zone within DRC

 - The description of the SPA as belonging to to UNFPA is misleading - while the citation does lead to a UNFPA webpage describing the SPA, SPA surveys were developed by and have always been conducted by the DHS program, first through Macro and then ICF. https://dhsprogram.com/

 - Donabedian provided a model for measuring health care quality, not health systems

 - Results can be simplified for readers by presenting only one of two quantities when there are only two categories (e.g. public vs private facilities, male vs female nurses)

Is the work clearly and accurately presented and does it cite the current literature?

Partly

If applicable, is the statistical analysis and its interpretation appropriate?

Not applicable

Are all the source data underlying the results available to ensure full reproducibility?

Yes

Is the study design appropriate and is the work technically sound?

Partly

Are the conclusions drawn adequately supported by the results?

Yes

Are sufficient details of methods and analysis provided to allow replication by others?

Partly

Reviewer Expertise:

Health system measurement, quality of maternal care, HIV prevention, implementation science

I confirm that I have read this submission and believe that I have an appropriate level of expertise to confirm that it is of an acceptable scientific standard, however I have significant reservations, as outlined above.

# Response

**Published:** 2026-04-01

**Authors:** Kieran Desmond

**Affiliations:** Not Applicable, UK

Thank you for your thorough and constructive feedback. The comments have led to significant structural improvements to the manuscript, particularly in the Introduction and Methods sections, and we believe the revised paper is substantially stronger as a result. We address each comment in turn below.  

**COMMENT **1 Introduction may be stronger with presentation of what a well functioning maternal and newborn care system looks like - e.g. capacity to do BEmONC and refer to facilities that can do CEmONC; specific inputs and processes to obtain desired patient outcomes - as well as the context of maternal health in DRC, and the similarity of Vanga to the rest of the country (including some of the setting information presented in methods). Results of prior health system assessments in DRC or (if applicable) any similar settings could be briefly mentioned. The details on how quality is measured and the suite of tools can be presented in the Methods section at the same time as this tool is defined. In other words, focusing the introduction on the What and Why questions and the Methods more on How would strengthen the article  

**AUTHOR RESPONSE **We have added a paragraph to the Introduction describing what a well-functioning maternal and neonatal care system looks like, including the BEmONC/CEmONC framework, the referral chain, and the key inputs required to achieve acceptable patient outcomes.   We have added a paragraph contextualising the DRC health system, including its structure, financing challenges and workforce shortages, and a separate paragraph summarising the findings of prior facility assessments in the DRC (Casey 2009, Casey 2015, Ntambue 2011, Mizerero 2021), establishing what is already known and the gap this study addresses.   Setting information previously in Methods 3.1 describing Vanga's geography, infrastructure challenges and economic context has been moved to the Introduction, with an argument for why Vanga is representative of rural DRC more broadly.   The description of the suite of assessment tools (EmONC framework, SPA, WHO SARA) and the Donabedian model have been moved from the Introduction to Methods 3.2, where they are presented alongside the definition of the tool used in this study. The definition of EmONC signal functions has been moved to Methods 3.3.

**COMMENT **2 Study design: the three types of data collection should not be described as domains - distinguish between the ‘what’ - the quality domains and indicators prioritized - and the ‘how’ - the type of data used to assess these domains. In clarifying this, please provide greater clarity on whether the Quality of Care domain crossed out in Figure 1 was excluded from the tool or was intended to be addressed using the caseload data; typically case load data is insufficient to provide insights on adherence to standards, beyond perhaps ‘partograph used.’ Additional detail on the types of records that were intended to be used and how this mapped to quality of care (if part of the original study design) would be useful. It would also be helpful to consolidate the description of the survey tool, which is described as based on the HHFA but also drawn from a separate French-language assessment. If this is described in one location and with clarification on if the French tool was an adaptation of the HHFA and how it differed from the original, that would assist the reader  

**AUTHOR RESPONSE **We changed the three data collection methods in methods section 3.2 to more clearly distinguish between the quality domains assessed “the what” and the data collection methods used to assess them “the how”. We removed the word domains when describing the data collection methods.  The description of the survey tools in methods 3.2 has been entirely re-written to clarify the use of the HHFA, the Habonimana 2022 tool and the DRC ministry of public health guidelines: it now reads in a single paragraph and outlines that the HHFA tool was the primary framework of the Habonimana questionnaire in french, and that the DRC guidelines were used only to contextualise for the local setting.  We expanded the footnote in figure 1 to clarify and justify why we were originally using caseload data to assess the quality of care domain (using proxy measures including complication rates and partograph use), and why, due to suspicions of widespread falsification, it was crossed out. Furthermore, we added detail to the part in methods 3.2 where we explain how caseload data was intended to assess quality of care.  We acknowledge the reviewer’s comment that more formal statistical analysis of the quality of data would strengthen the paper and we have noted this as a serious consideration for any future investigations of this type. We have included this weakness in the limitations section of the discussion, and outlined how it could have been improve with the formal statistical tests mentioned by the reviewer.   

**COMMENT **3 Several terms and classifications are used that have the effect of making the results less rather than more clear, including ‘type A’ and ’type B’ facilities as well as the grading system of indicators; in both cases, the underlying facts of the facilities and the indicator level are more useful than the labels. Why not use meaningful labels like BEmONC and CEmONC? Or doctor-led and nurse-led?  Whatever label, consider presenting the two tiers in the same order throughout - right now Type B comes first in the results but second elsewhere.  

**AUTHOR RESPONSE **The authors reclassified facility titles to “doctor-led” and “nurse-led” and restructured the ordering to make it easier for the reader to follow.   

**COMMENT **4 Level of analysis: the majority of the results are presented by indicator for the facility tier, as opposed to aggregating by facility to consider the readiness of each facility overall. This may be a reasonable choice given the goal of describing Vanga Zone as a whole (minus the referral hospital). This choice should be stated explicitly in the Methods; I would also consider summing up indicators within facilities as a way of providing insight on how much the facilities vary within the overall very low level of inputs to care identified.  

**AUTHOR RESPONSE   **We acknowledged more explicitly the choice to aggregate indicators across all facilities of that type, explaining that the justification was to most accurately reflect the study aim of assessing EmONC provision across the health zone. We state in the methods that we were concerned that displaying totals of indicator inputs for individual facilities may be beyond the limited scope of this initial study, as well as concerns over identification of the small number (5) doctor-led facilities.   

**COMMENT **5 Case load data and falsification: This is an important finding that is currently split between the methods and results, weakening its clarity and impact. The methods of case load data extraction and data quality assessment should be reported in the Methods section, and the findings and conclusion that the data are not reliable should be a key aspect of the Results section. For data quality checks, consider WHO data standards for HMIS as one resource https://www.who.int/data/data-collection-tools/health-service-data/data-quality-assurance-dqa, although these focus on too much variability rather than too little; other tests such as the Runs test to check for non-randomness may be helpful, as would reporting variance and plotting the data to show the patterns. It’s actually an interesting oversight in the data quality assurance process, and one that could make its own analysis, considering for instance expected variation in facility data as drawn from sources like SPA to inform standards. That’s beyond the scope of this paper, but I think a basic assessment of the data is well within the scope of this analysis and would be a valuable contribution.  

**AUTHOR RESPONSE **We worked to address the point that the caseload data and falsification finding was split between the results and methods by creating a clearer distinction between these two sections’ content. We added text to the Caseload data part of method section 3.2 outlining how our method would assess the quality of data: we would compare records across the two collection periods and look for identical or near-identical findings in numerous indicator categories,which we would consider indicative of possible falsification, after informal conversations with local staff to provide context to these findings.  In the results section 4.1 reporting the findings from nurse-led facilities we took the recommendation of the reviewer to include more rigorous defense of our suggestion of falsification. We did this by demonstrating and outlining the evidence of suspicious similarities between the two time periods of data collection. Specifically, we point to: median live births were 52 in both periods (P25 18-20, P75 66-67) and median normal deliveries were 51-52 across both periods (P25 18-20, P75 67). Median figures for caesarean sections, referrals and complications were equally consistent.  We also added a section to the discussion limitations section where we outline the reviewer’s point about including more rigorous statistical analysis like the Runs test to strengthen the argument.   

**COMMENT **6 The findings at present are quite granular, and could benefit from additional focus and synthesis to bring out key points. In addition to aggregating by facility, it may be helpful to consider use of the figures to illustrate the findings and highlight gaps. These could be basic column or bar plots to show frequency of the items found (ordered by frequency to highlight the least frequent) or heat maps / color coded tables to highlight gaps. See for example Yang et al, Antenatal corticosteroids in low- and middle-income countries (JOGH, 2025 doi: 10.7189/jogh.15.04149 ) for examples of data presentation that could be considered.  

**AUTHOR RESPONSE **We would like to thank the reviewer for this comment and wholly agree that the data visualisation suggestions mentioned would enhance the accessibility and impact of the findings. We chose to include the granular indicator-level data because our principal stated aim with this paper was to provide something that would inform policy-makers and local government actors to identify and implement targeted strategies to address specific issues, and we therefore felt that the most detailed data as possible was most appropriate. We tried to provide a summary-level analysis by using the Kosgei-adapted grading system across indicators with levels “excellent, good, moderate and poor” but we accept that this in fact detracts from clarity and, has been mentioned by another reviewer, is not a validated tool.  We agree that summary visualisations like those mentioned should have been presented in tandem with the granular form and that this would have strengthened the paper. We have made attempts to provide more statistical summary, such as in the revised section 4.1 where we present summary statistics of the caseload data similarities. In future studies, we will ensure that summary visualisations are included.   

**COMMENT **7 The results and discussion clearly draw attention to the major challenges in infrastructure within the Zone; can additional information be provided to contextualize this within DRC and suggest whether similar conditions are likely in other areas of the country? I understand this is a detailed assessment of all facilities within one zone and is not intended to represent the country as a whole, but some context to understand whether this zone is likely to be an extreme outlier or fairly similar to other rural areas would be helpful. Potentially outside the scope of this article, but understanding the time and financial cost of this assessment would be interesting in terms of considering what types of routine and in-depth assessment the country or regions within the country might undertake, given the extreme deficits identified and the need to prioritize resources for system strengthening.  

**AUTHOR RESPONSE **We amended the discussion section comparing existing literature to contextualise the findings from Vanga within the wider context of rural and remote DRC, citing similar studies conducted in the DRC, in which we were able to argue that the deficiencies identified are unlikely to represent outliers, instead being in-line with the situation across remote and rural DRC. We also amended the future directions section to include that in future studies of this nature, time and financial costs should be published to better inform funding bodies and policy-makers when making necessary budgetary decisions when commissioning future works of this nature.  

**COMMENT **8  Minor notes 1 Consider including a map to orient readers to Vanga Zone within DRC 2 The description of the SPA as belonging to UNFPA is misleading - while the citation does lead to a UNFPA webpage describing the SPA, SPA surveys were developed by and have always been conducted by the DHS program, first through Macro and then ICF. https://dhsprogram.com/ 3 Donabedian provided a model for measuring health care quality, not health systems 4 Results can be simplified for readers by presenting only one of two quantities when there are only two categories (e.g. public vs private facilities, male vs female nurses)  

**AUTHOR RESPONSE **  1. Generating sufficiently high quality, copyright-free maps of Vanga within the context of the wider DRC has proven difficult, efforts ongoing 2. We amended the text to clearly demonstrate the SPA surveys are conducted by DHS, and cite the relevant page on the DHS program website 3. We amended the reference to the Donabedian system to specify it is designed to measure healthcare quality, not health systems 4. Where applicable, this change has been implemented to remove unnecessary statistics for binary variables
